# Nanoencapsulated betulinic acid analogue distinctively improves colorectal carcinoma *in vitro* and *in vivo*

**DOI:** 10.1038/s41598-019-47743-y

**Published:** 2019-08-08

**Authors:** Debasmita Dutta, Brahamacharry Paul, Biswajit Mukherjee, Laboni Mondal, Suparna Sen, Chinmay Chowdhury, Mita Chatterjee Debnath

**Affiliations:** 1 Department of Pharmaceutical Technology, Jadavpur University, Kolkata, 700032 India; 2 Organic and Medicinal Chemistry Division, CSIR- Indian Institute of Chemical Biology, Kolkata, 700032 India; 3 Infectious Diseases and Immunology Division, CSIR- Indian Institute of Chemical Biology, Kolkata, 700032 India

**Keywords:** Nanoparticles, Cancer prevention, Molecular medicine

## Abstract

Betulinic acid, a plant secondary metabolite, has gained significant attention due to its antiproliferative activity over a range of cancer cells. A promising betulinic acid analogue (**2c**) with better therapeutic efficacy than parent molecule to colon carcinoma cells has been reported. Despite impressive biological applications, low aqueous solubility and bioavailability create difficulties for its therapeutic applications. To overcome these lacunae and make it as a promising drug candidate we have encapsulated the lead betulinic acid derivative (**2c**) in a polymeric nanocarrier system (**2c-NP**) and evaluated its *in vitro* and *in vivo* therapeutic efficacy. Apoptosis that induces *in vitro* antiproliferative activity was significantly increased by **2c-NP** compared to free-drug (**2c**), as assured by MTT assay, Annexin V positivity, JC1 analysis and cell cycle study. The therapeutic potential measured *in vitro* and *in vivo* reflects ability of **2c-NP** as an effective therapeutic agent for treatment of colon carcinoma and future translation to clinical trials.

## Introduction

**Figure 1:**
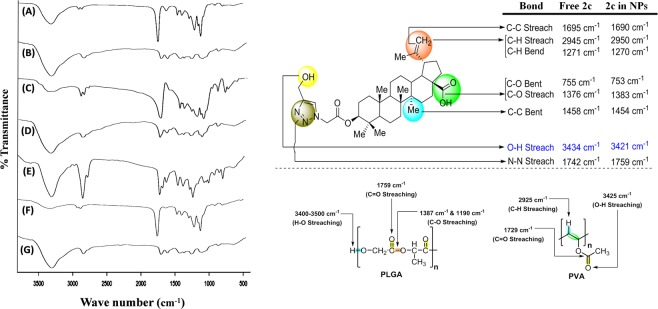
Fourier transforms infrared spectral analysis. (A) Blank nanoparticles (without drug); (B) Polyvinyl alcohol (PVA); (C) polylactide-co-glycolide (PLGA); (D) Mixture of PLGA and PVA; (E) Drug, Lead Betulinic acid analogue, **2c**; (F) Nanoparticles loaded with **2c**; (G) Mixture of PLGA, PVA and **2c**. Schematic representation of bond streaching of 2c, PLGA and PVA.

Colon cancer, the neoplastic condition of large intestine, is associated with significant morbidity and mortality^1,2^. Conventional chemotherapeutic agents are the mainstay of treatment strategies apart from surgical resection^3^. However, the high toxicity of these agents towards normal cells and a continuous increase in drug-resistance of neoplastic cells demand development of new chemical entities with site specific delivery. In this regard, plant sources were explored extensively in last few decades in search of new chemical entities. Betulinic acid (BA), a member of pentacyclic triterpene class, has been shown to possess potent cytotoxic effects against an array of cancerous cells originated from liver, colon, lung, ovarian, cervical, and neuroblastoma *in vitro*^4,5^, with a high dose tolerability (up to 500 mg/kg body weight)^6^. Due to its hydrophobic nature many attempts were made to develop new derivatives. Earlier we reported a new library of betulinic acid analogues^7^, out of which compound **2c**, possessing a 1,2,3-triazole moiety attached to C3 hydroxy group of BA through a linker (Supplementary Fig. S1), proved markedly more efficacious than BA and the other analogues against colon cancerous cells (i.e., HT-29). It was capable of inducing two different types of programmed cell death (PCD), namely, apoptosis and autophagy^7,8^ in HT-29 cells. However, its solubility leading to poor systemic absorption remained a serious concern for its emergence as a potential drug candidate^9^. We have now developed a nanoformulation of **2c (For structure see Fig.** 1) based on the biodegradable polymer PLGA which has the ability to allow hydrophobic drugs to enter into and sustain in the tumor area through enhanced permeability and retention (EPR) effect^10^. The designed nanoformulation was assessed for its cytotoxic potential *in vitro* in human colorectal cancer cells, HT-29. Bioavailability and tumor specificity of this nanoformulation was confirmed by *in vivo* biodistribution studies. The *in vivo* antitumor efficacy of **2c-NP** was evaluated in mice and rats bearing colorectal cancer. The study was conducted with the hypothesis that the proposed nanoparticulate delivery system containing the BA analogue (**2c-NP**) would enhance therapeutic payload at the target site to combat with the severity of this deadly disease. This may provide us a novel and safer therapeutic alternative to the standard chemotherapeutic regimen of colorectal cancer.

## Results

### Study of drug-excipient interactions by fourier transforms infrared spectroscopy (FTIR)

FTIR spectrophotometric investigation was used to determine the interaction between bioactive chemically modified **2c** and the excipients used for nanoformulation. Spectra of the betulinic acid derivative (**2c**), each individual excipient (such as PLGA and PVA), their physical mixture and formulation with or without drug, were recorded (Fig. 1). The characteristic peaks of the triazole linked betulinic acid derivative at the wave numbers 3434 (O-H stretching of free -OH group), 1376 (C-O stretching of –COOH group), 2945 (C-H stretching of C_sp_^2^-H bond of –CH_2_ group) and 1271 (C-H bending of C_sp_^2^-H bond of –CH_2_ group) cm^−1^ were present. All of the characteristic bands of PLGA and **2c** were found in the FTIR spectrum of their physical mixture. Only the characteristic peaks of the polymer were found in the spectrum of nanoparticles without drug (**2c**). Some minor peak shiftings of formulation ingredients were noted, attributed to the presence of some weak physicochemical interactions such as van der Waals force of attraction, formation of weak H-bonds, dipole–dipole or dipole–induced dipole interaction, etc. which may play important roles in formation of spherical shape of the nanoparticles. The peaks obtained from drug loaded formulation were almost similar with those obtained from blank formulation. This suggests absence of free-drug on the surface of nanoparticles^11^.

### Characterization of 2c-NP

#### Evaluation of particle size, size distribution and zeta potential

We next studied the physical characteristics of PLGA formulated nanoparticles, **2c-NP**. The size as obtained from dynamic light scattering (DLS) method ranged from 80 nm to 750 nm with an average of 380 nm (Supplementary Fig. S2). The zeta potential of **2c-NP**, which is a measure of the average surface charge of drug encapsulated nanoparticles, was recorded as −8.94 ± 1 mV. This is within the range of −30 to +30 mV suggesting its instability in long term standing condition in aqueous medium. Hence it should preferentially be preserved as powder, to be dissolved in medium before administration^11,12^.

#### Estimation of percentage drug loading and drug loading efficiency

The loading percentage of drug was estimated as 8 ± 0.2% for **2c-NP**. Drug loading in the delivering vehicle determines the amount of formulation required for administration, an important factor to understand the therapeutic ability of the drug delivery system^11^. The encapsulation efficiency of **2c-NP** was found to be 86%, showing that the method is efficient enough to prepare nanoparticles with little loss of materials.

#### Study of surface morphology

**Figure 2:**
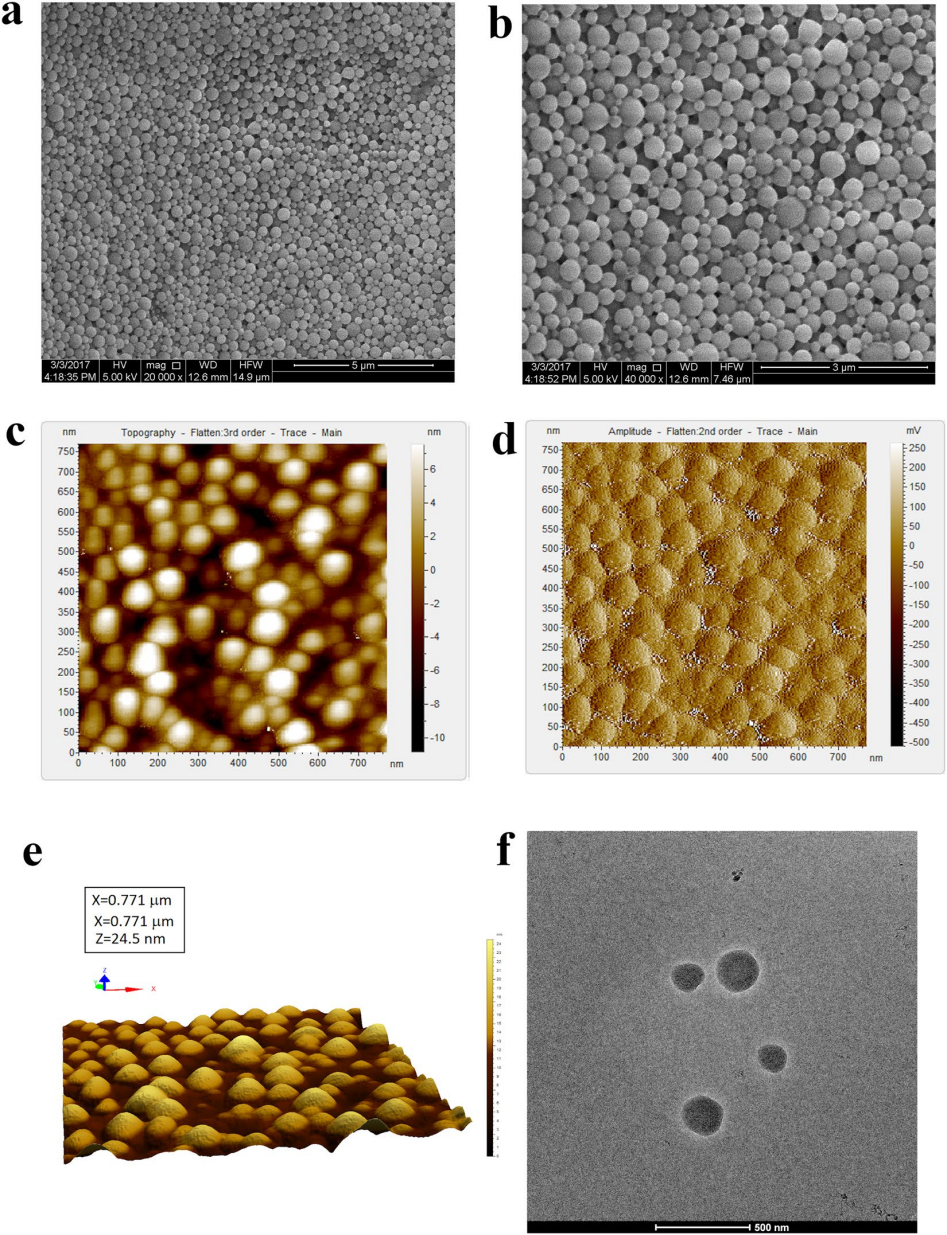
Morphological analysis of 2c-NP. (**a**,**b)** Field emission scanning electron microscopy images of **2c-NP**s under JEOL microscope (JSM-7600F) at 20,000× and 40,000× magnification respectively. **(c)** Topography, **(d)** Amplitude and **(e)** 3D view of atomic force microscopy (AFM) image of **2c-NP**. **(f)** Cryo- TEM image of **2c-NP**s at ~80,000× magnification.

Surface morphology of the formulated nanoparticle plays a crucial role in drug uptake by cells or organs as initially the nanoparticle surface interacts with biomembrane and this influences its distribution and effects. Surface morphology of **2c-NP** was primarily observed by FESEM (Fig. 2a,b). The images unveiled that formulated nanoparticles were mostly spherical in shape in a nano size range of 130 nm to 350 nm with maximum particles of 250 nm size. The high resolution data confirmed the smooth surface of the nanoparticles without any visible pores or cracks on the surface, which suggests mechanical/structural rigidity. AFM images (Fig. 2c–e) revealed absolute spherical shape with smooth surface. It showed a closely packed arrangement of spherical nanoparticles with smooth surface. 3D image (Fig. 2e) of AFM shows well-separated nanoparticle with a narrow size distribution. Cryo-TEM study describes the internal structure of the experimental nanoparticle. Cryo-TEM images (Fig. 2f) demonstrate dark black stain throughout the nanoparticle which ensures homogenous drug distribution in the nanoformulation.

#### *In vitro* drug release study of 2c nanoparticle (2c-NP)

**Figure 3:**
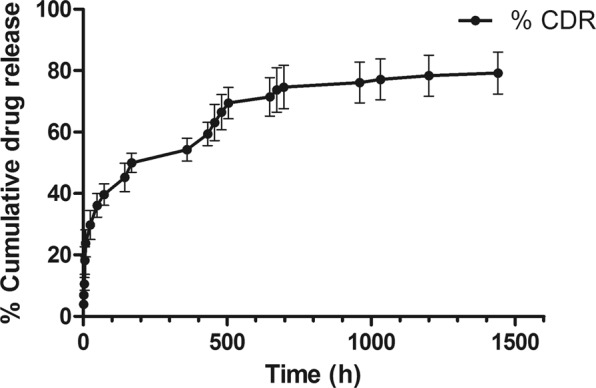
Cumulative % drug release from **2c-NP** nanoparticle against Time. Data show Mean ± SEM of experiment in triplicates.

Drug-release kinetic study of the prepared nanoparticle was conducted (Fig. 3) and the data were tested by zero order, first order, Hixson–Crowell, Korsmeyer–Peppas, and Higuchi kinetic models. Supplementary Table T2 describes the regression coefficient values (R^2^) of different drug-release kinetics of **2c-NP**. The data suggest that the release kinetics of the prepared nanoformulation was in closest proximity with Higuchi model as evaluated by the regression coefficient value (R^2^), 0.9209. This indicates that drug release from the nanoparticle matrix involves simultaneous penetration of the surrounding liquid into the matrix, dissolution of the drug, and diffusing out through interstitial channels or pores based on volume and length of the opening^13^.

#### Cell viability assay

**Figure 4:**
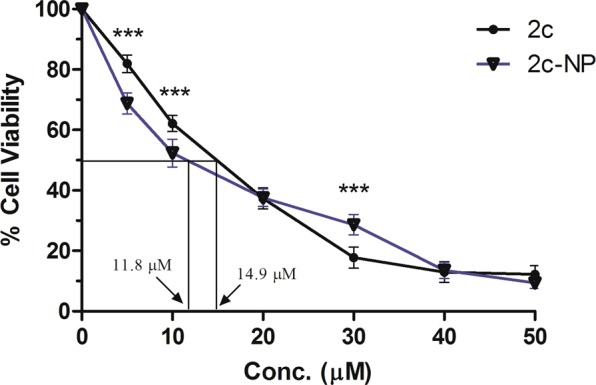
MTT assay for assessment of *in vitro* cytotoxicity. *In vitro* cytotoxicity of **2c**, and **2c-NP** was evaluated on HT-29 cells. IC_50_ values are marked for comparative analysis. Data points are represented as mean ± SEM of three individual experiments. Statistical analysis of data was conducted using one-way ANOVA and Tukey’s post hoc test using GraphPad Prism (version 5.0) software (California, USA) (*P < 0.05 was considered as the statistical level of significance, when compared the data of 2c-NP with those of 2c).

In our earlier work we determined the cytotoxic effect of betulinic acid analogue (**2c**) over a range of concentrations (0–50 µM) for up to 48 h and found that analogue **2c** exhibited IC_50_ value, 14.9 µM on HT-29 cells^7^. To enhance its bioavailability, avoid loss of free-drug and to lower its cytotoxicity towards normal cells, we prepared **2c** encapsulated PLGA nanoparticle (**2c-NP**). The IC_50_ value of **2c-NP** on HT-29 cells was found to be 11.8 µM, significantly lower than the IC_50_ value of free-drug (Fig. 4). Furthermore, we investigated the antiproliferative activity of **2c-NP** on other colorectal cancer cell lines such as HCT-116 and HCT-15 (Supplementary Table T1). **2c-NP** deciphered negligible cytotoxicity toward normal kidney cell line, HEK 293 and normal colon cell line CCD-33-C_O_ (Supplementary Table T1). As **2c-NP** was found to exhibit the highest cytotoxicity toward HT-29 cells, further *in vitro* studies were investigated primarily on HT-29 cells.

#### Cellular uptake study

**Figure 5:**
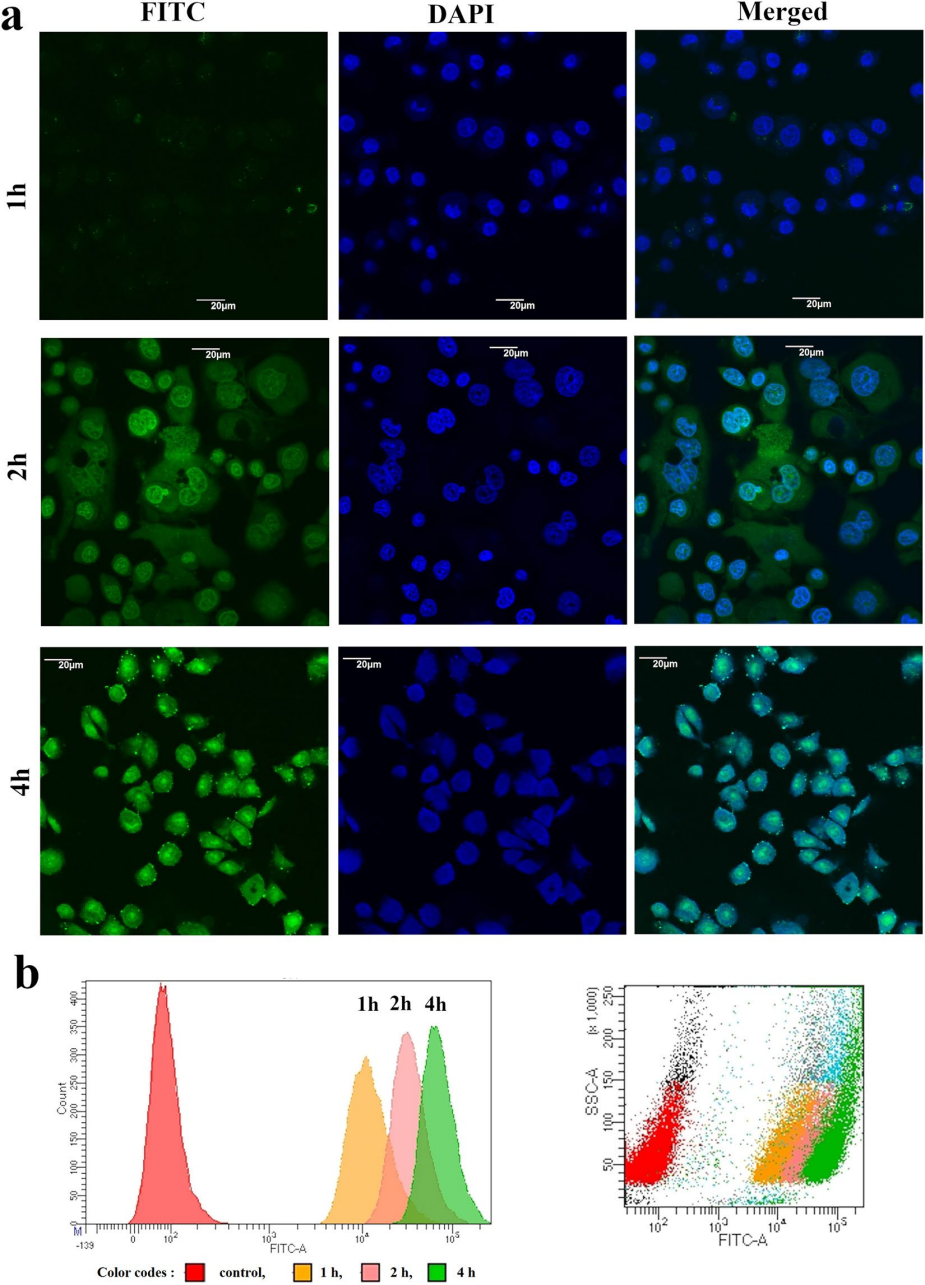
Cellular uptake study. (**a**) Confocal microscopy images of HT-29 cells after treating with FITC-**2c-NP** for 1 h, 2 h and 4 h respectively. Scale bar represents to 20 µm. (**b**) Flow cytometry analysis representing distribution of FITC-**2c-NP**s in HT-29 cells after treating them for 1 h, 2 h and 4 h at 11.8 µM concentration.

*In vitro* cellular uptake of nanoparticles was evaluated by confocal microscopy and quantified by FACS analysis using FITC-labeled PLGA nanoparticle encapsulated **2c** (FITC-**2c-NP**). Figure 5a depicts confocal microscopy images of HT-29 cells following incubation with FITC-**2c-NP** for 1, 2, and 4 h. At 2 h, FITC-**2c-NP** was found to accumulate in the cells more particularly distributed in the cytoplasm, not the nuclei. The intensity of green fluorescence near nuclear region of cells at 4 h showed the ability of nanoparticles to reach in nucleus. All the images demonstrated time dependent endocytosis of nanoparticles in HT-29 cells. Flow cytometric analysis, further, showed a robust increase in median fluorescence intensity, indicating a consistent increase in intracellular uptake of FITC-**2c-NP** (Fig. 5b). Both the confocal and flow cytometric studies revealed that FITC-**2c-NP** was predominantly endocytosed in HT-29 cells^14^.

#### Detection of cell death by acridine orange/ethidium bromide dual staining

**Figure 6:**
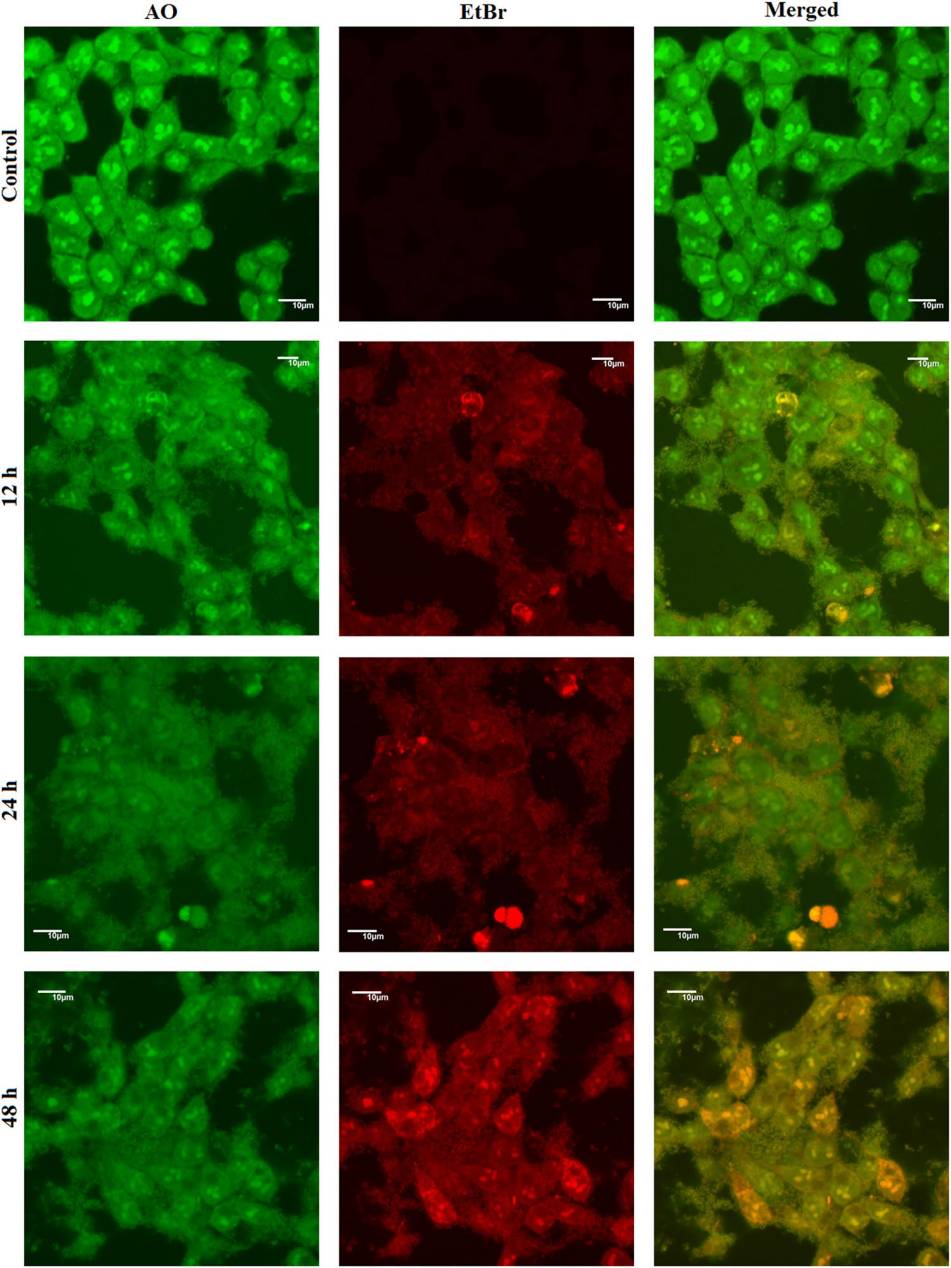
Acridine Orange/Ethidium Bromide (AO/EB) dual staining. Confocal microscopy images of HT-29 cells after treating with **2c-NP** (11.8 μM; 0–48 h) followed by staining with acridine orange (4 µg/ml) and ethidium bromide (4 µg/ml) as observed under Olympus Fluoview 10i confocal microscope at 100X magnification. Scale bar represents 10 µm.

When observed under confocal laser microscope the control cells appeared as green with no orange or red spot, indicating the live and healthy condition of the cells (Fig. 6, panel ‘control’). Treatment with **2c-NP** for 12 h, caused appearance of orange nucleus in a significant population of cells. As the treatment time increased to 24 h the number of orange cells increased and some cells turned reddish indicating cell death. On higher zooming and changing the laser distinct orange spots were observed in the cytoplasms of some cells (shown in Supplementary Fig. S3), indicating production of acidic vacuoles^15^. At 48 h the number of apoptotic cells (yellowish orange to orange) increased in a large proportion. This result also correlates with the outcome of apoptosis analysis by FACS.

#### Mitochondrial membrane depolarization analysis using JC-1

**Figure 7:**
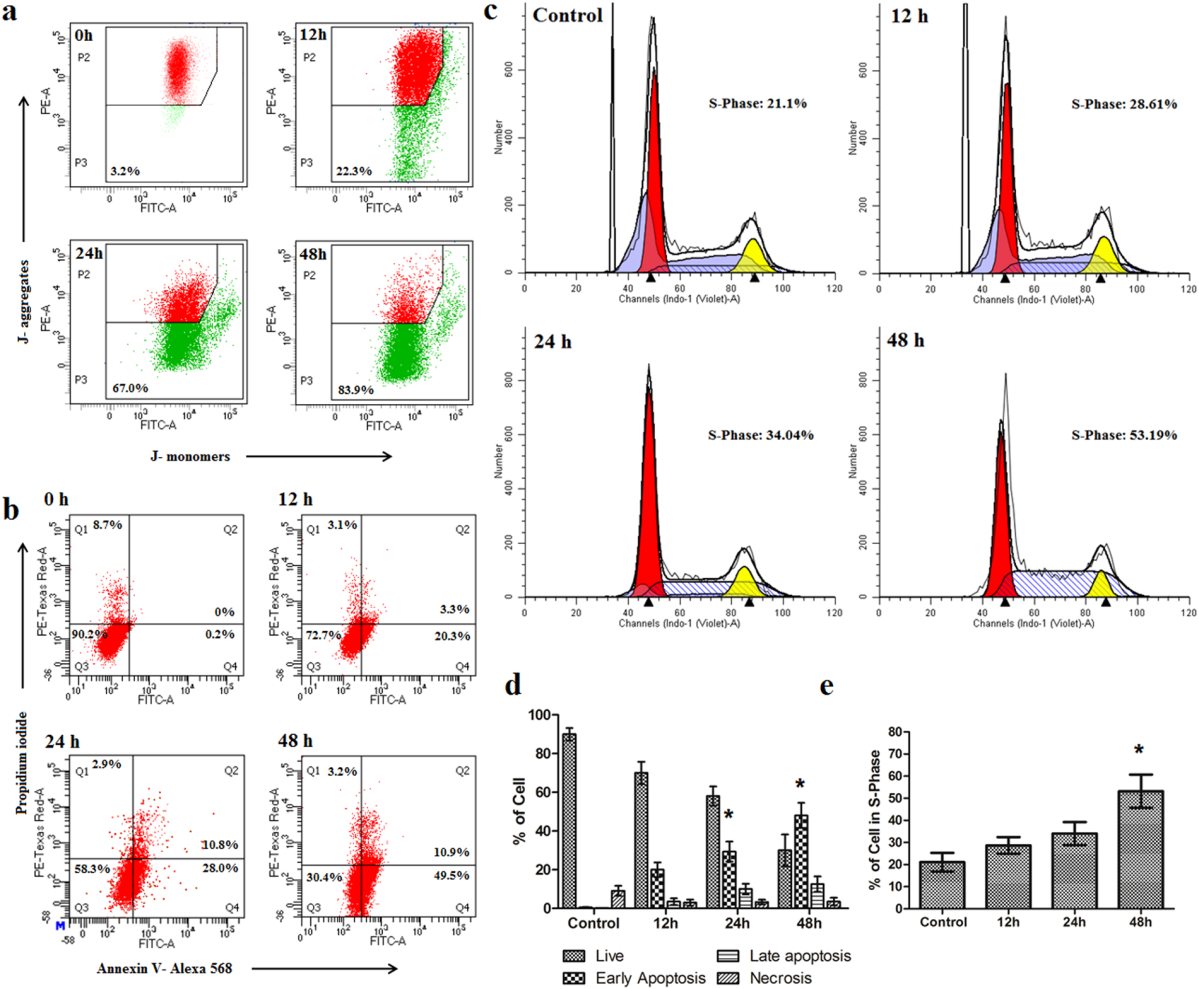
Mitochondrial membrane depolarization, apoptosis and cell cycle analysis study in HT-29 cells. (**a**) Loss of mitochondrial membrane potential in HT-29 cells as estimated by JC-1 analysis after treating cells with **2c-NP** (11.8 μM) for 12 h, 24 h and 48 h. (**b**) Induction of apoptosis in HT-29 cells by **2c-NP** (11.8 μM) after treating them for 12 h, 24 h and 48 h. **(c**) Cell cycle analysis of HT-29 cells after treating them with **2c-NP**s. **(d**) Quantification of apoptosis assay (Annexin V/PI) and cell cycle analysis. Data shown in bar diagram are average of the three individual experiments represented as mean ± SEM. Statistical analysis of data was carried out using one-way ANOVA and Tukey’s post hoc test using GraphPad Prism (version 5.0) software (California, USA) (*P < 0.05 indicates statistical level of significant time points that bring considerable changes in S-phase of cell cycle and early apoptosis with respect to control).

One of the important indicators of apoptosis is a loss of mitochondrial membrane potential. The mitochondrial membrane potential was measured by staining the cells with JC-1, a highly lipophilic, cationic dye which is capable to selectively enter in mitochondria and acts as a dual emission probe^7^. In normal cells, mitochondrial membrane potential is highly negative, so JC-1 rapidly enters in mitochondria where it forms aggregates. These aggregates emit red fluorescence (595 nm) when excited at 490 nm. Thus, JC-1 monomer/aggregate ratio (535 nm/595 nm ratio, also known as green/red ratio) increases in apoptotic condition. Our previous experiments on HT-29 cells treated with free-drug, **2c**, showed 16.5% of green fluorescence at 12 h, 30.5% at 24 h and 51.4% at 48 h with respect to normal cells^7^. When we treated HT-29 cells with IC_50_ concentration of **2c-NP**, the percentage of cells with depolarized mitochondria increased to 67% at 24 h and 83.9% at 48 h (Fig. 7a). The highest value of JC-1 monomer/aggregate ratio (sign of mitochondrial membrane depolarisation indicating apoptosis) was obtained at 48 h of treatment of with **2c-NP**.

#### Apoptosis assay

To investigate the capability of **2c-NP** to induce apoptosis in HT-29 cells, the treated HT-29 cells were incubated with Alexa Fluor^TM^568 labeled Annexin V and propidium iodide (PI)^16^. FACS analysis showed that apoptosis induction increased with time. According to our previous report, Annexin V binding to HT-29 cells following treatment with the lead compound, **2c** was detected 16.1% at 12 h followed by 20.3% and 30% at 24 h and at 48 h respectively^7^. After 12 h, 24 h and 48 h of **2c-NP** treatment, 23.6%, 30.8% and 60.4% Annexin V bindings to HT-29 cells were found respectively (Fig. 7b). The findings suggested that **2c-NP** predominantly enhanced externalization of phosphatidylserine, indicative of apoptosis, compared to free-drug, **2 c**^7^.

#### Analysis of cell cycle

The cell cycle regulatory pathway is of prime importance in tumor development and progression. To postulate the exact point of cell cycle arrest we quantified DNA content in cells after staining with propidium iodide. When treated HT-29 cells with **2c-NP** a number of significant changes was observed in the cell cycle pattern compared to the untreated cells (Fig. 7c). A descriptive file of cell cycle analysis by ModFit LT 5.0 has been shown in Supplementary File (Supplementary Fig. S5). From the figure of FACS analysis (Fig. 7c) and bar diagram (Fig. 7d) we can conclude that a time dependent arrest happened to S-phase of the treated cells. The cell population increased in S-phase with increasing treatment time, with cells passing from G1-phase to S-phase while DNA synthesis stopped, showing a block at S-phase. This observation is also supported by the earlier literature reports on cytotoxic effect of BA on CL-1 and D17 cells^17^. Furthermore, a slight increase in G2/M ratio occurred due to an increase in treatment time. This finding implies that cells are blocked in interphase (G2) promoting arrest of mitotic cell division after **2c-NP** treatment. The underlying mechanism of blocking at the cell cycle, appears to be chromosomal damage that could not be repaired and committed to programmed cell death. For further confirmation of this hypothesis, we have performed a chromatin condensation assay after treating HT-29 cells with **2c-NP**.

#### Quantification of DNA degradation

Hoechst stains are cell permeable DNA specific dyes often used for identification DNA damage. After staining **2c-NP** treated HT-29 cells with Hoechst 33258 we found that amount of double stranded DNA was reduced with increase of treatment time (Supplementary Fig. S4), thus indicating degradation of DNA upon **2c-NP** treatment^8^.

#### p53 expression and caspase assay in different colon cancer cell types

**Figure 8:**
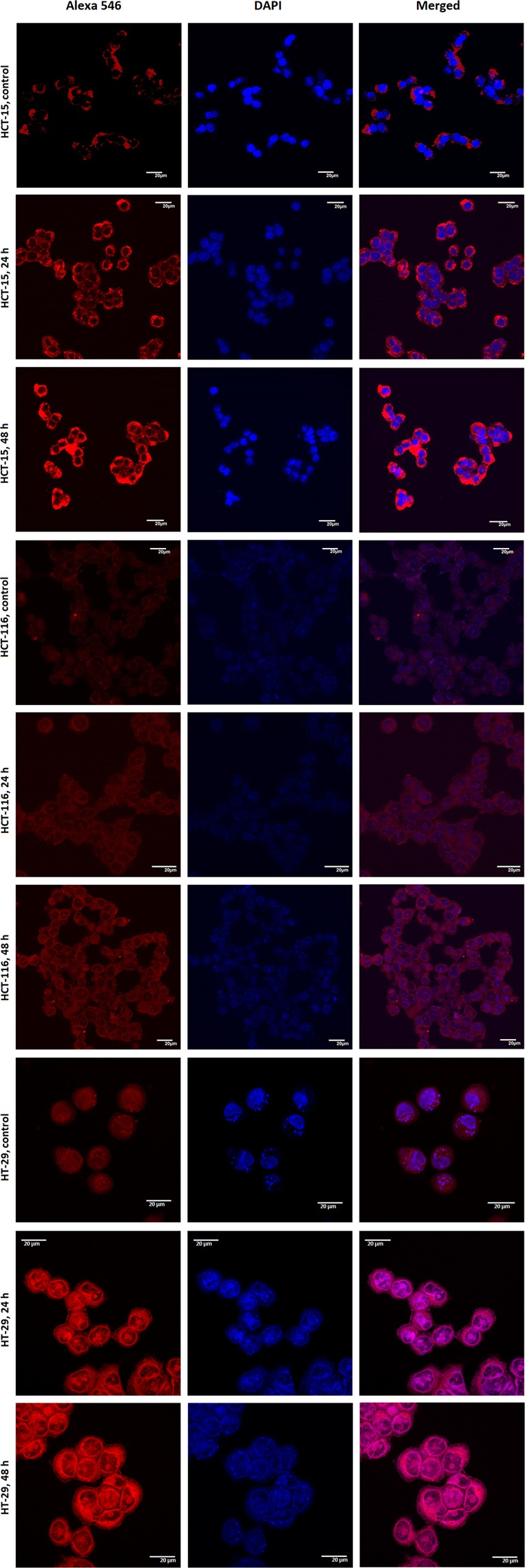
Expression of p53 in untreated HCT-15, HCT-116 and HT-29 cells (control panels) and upon exposure to **2c-NP** for 24 h and 48 h. Cells were labelled with rabbit anti-p53 primary antibody and further stained with Alexa Fluor-546 coupled goat anti-rabbit IgG secondary antibody. Panels: Left- Alexa Fluor -546 represent p53, Middle- DAPI representing nucleus and Right- merge of them.

To study the mechanism of cell death induction by 2c-NP in different colorectal cancer cells, p53 expression level was evaluated by confocal microscopy as p53 expression can predict apoptosis or necroptosis in cells^18^. Upon exposure to **2c-NP**, HT-29 cells showed the highest level of p53 expression compared to HCT-15 and HCT-116 cells (Fig. 8). The expression of p53 may also be due to necroptosis instead of apoptosis^18^. p53, when activated can induce intrinsic pathway of apoptosis by transactivating pro-apoptotic genes such as *BAX*, *BAD*, *Bak*; and also by directly binding with mitochondrial anti-apoptotic proteins such as Bcl-2, Bcl-XL, etc. p53 induced intrinsic pathway of apoptosis which is associated with upregulation of caspases protein family. Apart from this, p53 can induce necroptosis directly by opening mitochondrial membrane permeability transition pore (MPTP) or indirectly by upregulating necrosis-related factor (NRF), which (NRF) has an important role in controlling RIPK1/RIPK3 (Receptor-interacting serine/threonine-protein kinase 1 and 3) levels, the two important proteins responsible for necrosome formation. Activation of caspase 8 causes cleavage of RIPK1 and RIPK3 which in turn associated with necroptosis blockage^18^. Hence, we analysed caspase-3, -9, and -8 expression level in HT-29 cells after incubation of **2c-NP** for 12 h, 24 h and 48 h using colorimetric assay. An exponential increase in the activity of caspase-9 (∼12.6 fold increase compared to control) and caspase-3 (∼5.5 fold increase compared to control) was observed up to 6 h after which the activity reached a plateau phase. However, for caspase-8 the exponential increase (∼4.3 fold increase compared to control) was observed up to 6 h (Supplementary Fig. S6). Data suggests that cells would go more towards apoptosis avoiding necroptosis.

#### Biodistribution study in animal model

Colorectal cancer was developed in both mice (Swiss albino) and rats (Sprague Dawley) (SD) and biodistribution of **2c-NP** was assessed using ^99m^Tc radiolabeled **2c-NP** at different time points. Table 1 demonstrates biodistribution pattern of radiolabeled **2c-NP** in different organs/tissues of animals. The values for different organs are represented as percentage of injected dose (%ID) in whole organ/tissue except blood and muscle; expressed as percentage of injected dose per gram of tissue (%ID/g) (Table 1) and calculated as follows

1 $$ \% {\rm{ID}}=\frac{Radioactivity\,present\,in\,an\,organ}{Total\,radioactivity\,injected\,in\,blood}\times 100$$

2 $$ \% {\rm{ID}}/{\rm{g}}=\frac{Radioactivity\,present\,in\,an\,organ}{Total\,radioactivity\,injected\,in\,blood\times Weight\,of\,the\,organ}\times 100$$

**Table 1:** Biodistribution of ^99m^Tc-2c-NP in Sprague Dawley rats and CRC Swiss albino mice with colorectal cancer.

Organ/Tissue	%ID of ^99m^Tc-2c-NP in CRC rats (SD)			%ID of ^99m^Tc-2c-NP in CRC mice (SA)		
	1 h	2 h	5 h	1 h	2 h	5 h
Blood*	2.138 ± 0.121	1.710 ± 0.094	0.809 ± 0.065	3.266 ± 0.055	2.698 ± 0.024	1.489 ± 0.015
Heart	1.522 ± 0.008	1.666 ± 0.014	1.717 ± 0.023	0.542 ± 0.038	0.481 ± 0.024	0.372 ± 0.042
Liver	42.84 ± 4.121	36.81 ± 5.368	26.66 ± 2.112	28.42 ± 2.162	21.14 ± 1.424	17.26 ± 1.722
Lungs	1.104 ± 0.092	1.780 ± 0.063	1.910 ± 0.024	1.000 ± 0.162	1.393 ± 0.123	1.660 ± 0.054
Stomach	1.322 ± 0.151	1.226 ± 0.096	1.369 ± 0.110	0.988 ± 0.151	1.246 ± 0.082	1.404 ± 0.120
Spleen	1.664 ± 0.101	1.824 ± 0.091	1.962 ± 0.072	1.892 ± 0.113	3.464 ± 0.288	4.114 ± 0.352
Intestine	3.042 ± 0.388	5.448 ± 0.741	6.101 ± 0.527	3.942 ± 0.164	4.155 ± 0.387	5.024 ± 0.671
Kidney	6.758 ± 1.102	7.134 ± 0.858	5.722 ± 0.761	8.758 ± 1.008	6.226 ± 0.716	4.175 ± 0.328
Urine	16.44 ± 2.743	21.23 ± 2.452	29.21 ± 4.52	10.44 ± 1.743	16.23 ± 1.826	26.45 ± 2.110
Muscle*	0.047 ± 0.009	0.062 ± 0.005	0.051 ± 0.010	0.051 ± 0.006	0.053 ± 0.008	0.071 ± 0.009

Results are expressed as percentage of injected dose per organ/tissue except blood and urine where it is expressed as percent injected dose per gram (marked as*). Each value represents mean ± SEM of five animals per group.

Accumulation of **2c-NP** in colon reached highest at 5 h (compared to other time points studied). A significant portion of **2c-NP** accumulated in liver within one hour but was gradually eliminated with the progress of time. The major elimination of radiolabeled **2c-NP** occurred through urinary pathway. Negligible change in **2c-NP** accumulation in stomach with time indicates *in vivo* stability of the formulation^19^. As exact localisation and separation of tumor/cancerous part from colon proved difficult, the tumor specificity could be ascertained by comparing ratio of %ID/g of intestine and muscle. This ratio was found to be maximum with a value 11 in case of rats and 13 in case of mice, indicating preferential tumor selectivity of **2c-NP**.

#### *In vivo* gamma scintigraphic analysis in mice and rats with colorectal cancer

**Figure 9:**
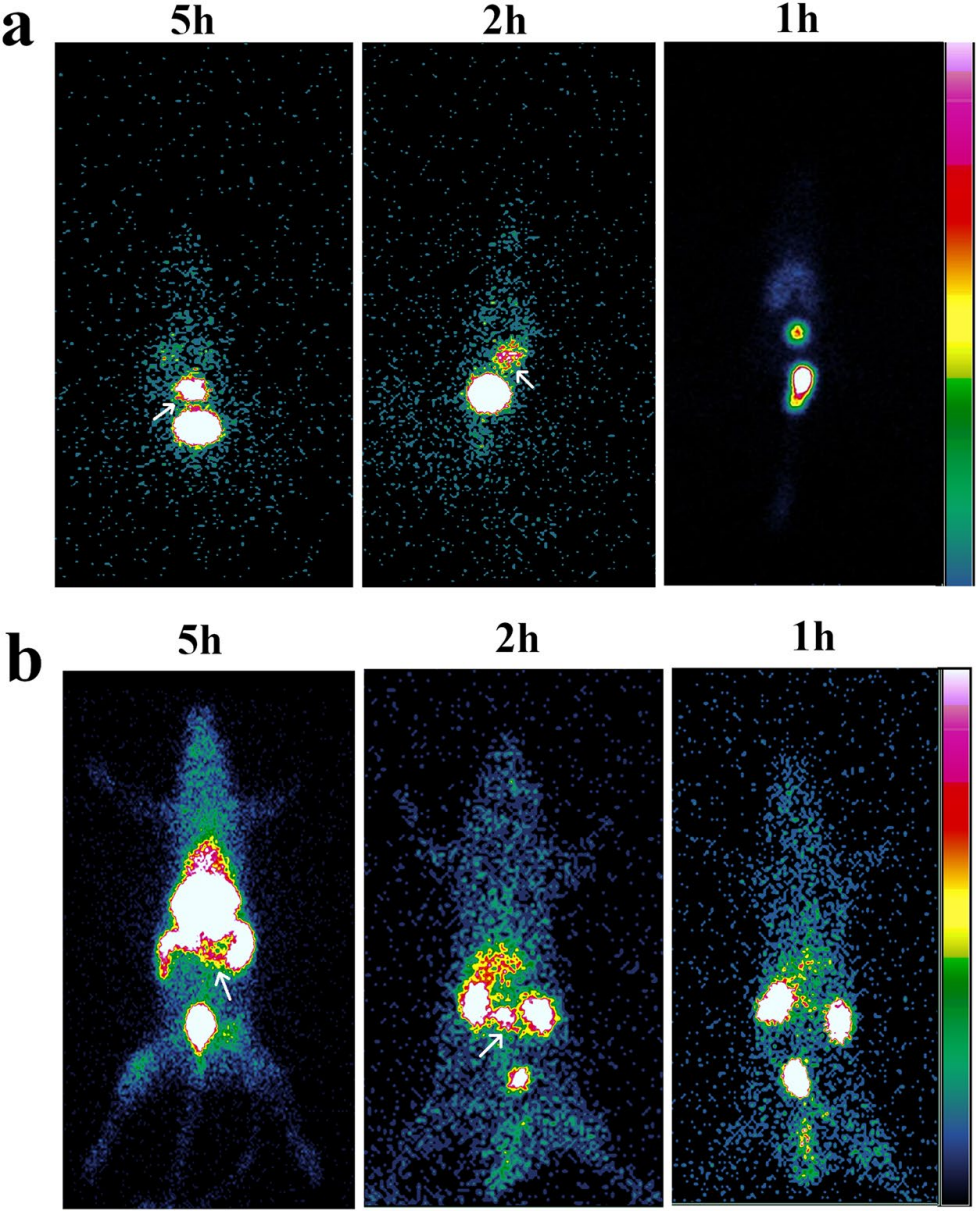
*In vivo* scintigraphic imaging. (**a**) Gamma scintigraphic images of Swiss albino mice at 1 h, 2 h and 5 h after treating them with ^99m^Tc – radiolabeled **2c-NP**. (**b**) Gamma scintigraphic images of Sprague Dawley rats at 1 h, 2 h and 5 h after treating them with ^99m^Tc – radiolabeled **2c-NP**.

The images acquired reveal a well-defined distribution of radiolabeled **2c-NP** in colon of tumor bearing mice and rats. A remarkable quantity of **2c-NP** deposited in the liver of rats and mice along with colonic regions also [Fig. 9a (for mice), 9b (for rats)]. A significant quantity of radiolabeled **2c-NP** also accumulated in colonic region of both mice and rats. Figure 9b demonstrates selective accumulation of **2c-NP** in both transverse and distal colon of rats, which may also denote generation of tumor in those regions of colon due to treatment with DMH. As the radioactivity distributes mostly in the abdominal cavity it was difficult to identify the exact position of tumors in colon from the scintigraphic images of live animals. So, to ensure accumulation of radiolabeled **2c-NP** in colon, we isolated the colons from the rats and mice, placed on petridishes and measured the radioactivity present in isolated colons under gamma camera (Supplementary Fig. S7b,d) and removed the portions of the tissues. The radioactivity detected from the tissues at different time points correlates with that found from the live animals (Supplementary Fig. S7a,c). The comparative intensities of radiation captured in gamma camera from different body parts also fairly matched with outcome of the bio-distribution studies. Further, those removed parts of the tissues were processed for histopathological investigations which confirmed that the removed tissues had cancerous growth (Supplementary Fig. S7b,d).

#### *In vivo* colonic distribution of FITC-2c-NP

**Figure 10:**
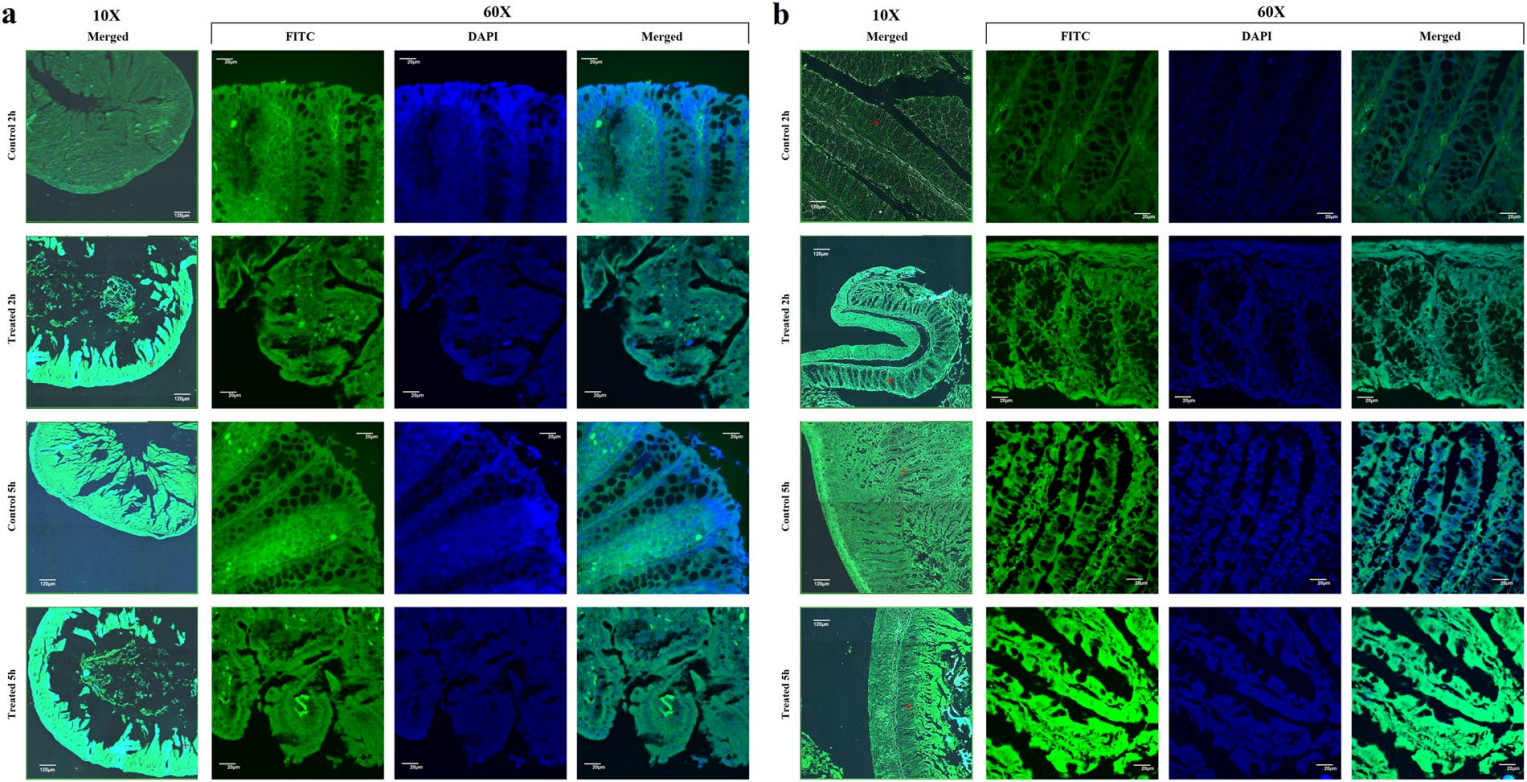
*In vivo* tissue imaging. Confocal microscopic images of cross section of (**a**) mice, (**b**) rat colon (normal/control and DMH treated), at 2 h and 5 h post-injection of FITC loaded **2c-NP** administration. Images are shown at 10× (**a**,**b**), and 60× (**a**,**b**) magnifications. Scale bar used at 10× magnification panel represents 120 µm and for 60X, 20 µm.

Confocal microscopy images reflected a wide distribution of FITC labeled **2c-NP** throughout the colon (Fig. 10). A significant accumulation was observed in both normal and affected (CRC model) colon epithelium, even in the distal ends of colonic crypts^20^. In case of rats with colorectal carcinogenesis colon accumulation of FITC-**2c-NP** was distinctly higher in some specific regions, which suggests that nanoparticle formulation significantly enhanced the endocytosis of it in colorectal cancer cells probably due to EPR (enhanced permeability and retention) effect^21^. Fluorescence intensity after 5 h post injection was found to be markedly higher than 2 h. This implies a time-dependent accumulation of **2c-NP** in target organ (the colon). The fluorescent images of normal colon showed no significant morphological or cellular alterations ensuring absence of detrimental effects on the normal tissue upon the experimental treatment.

#### Histopathological study of colon of normal animals, DMH treated animals and DMH treated animals treated with 2c-NP

**Figure 11:**
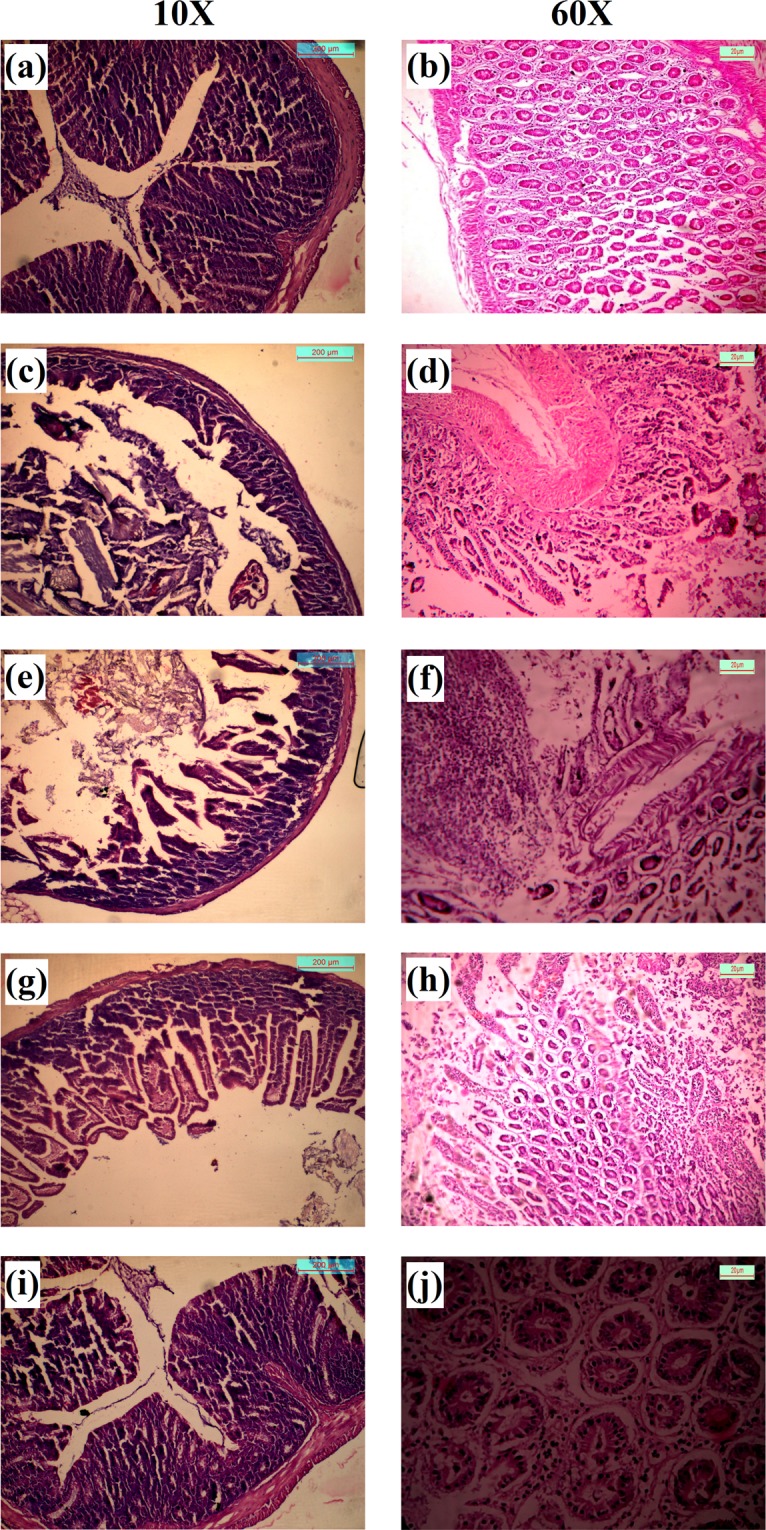
H&E staining of mice colon. (**a**,**b**) Normal colon at 10× and 60× magnifications; (**c**,**d**) Initiation of colon adenocarcinoma after treatment with DMH for a 7 weeks (at 10× and 60× magnifications respectively); (**e**,**f**) Development of colon adenocarcinoma after completion of treatment regimen with DMH (12 weeks) (at 10× and 60× magnification respectively); (**g**,**h**) Improvement of cancerous region after completion of treatment of colorectal cancer mice treated with **2c-NP** (9 weeks) (at 10× and 60× magnifications respectively). **(I**,**j)** Normal colon after treatment with **2c-NP** (at 10× and 60× magnifications respectively). Scale bar used in 10× and 60× magnification panels represent 200 µm and 20 µm respectively.

Cross sections of three different types of colon (normal, dimethylhydrazine treated CRC animals and **2c-NP** treated CRC animals) were stained with hematoxylin and eosin, and observed under optical microscope (Fig. 11). Upon close observation uniform layers of mucosal and submucosal epithelial cells were observed in normal mice colon. Side by side arrangement of healthy goblet cells was present in the epithelial layer, which was more prominent in confocal microscopy images at higher magnification (Fig. 10). The smooth muscle of colon also appeared thick, healthy and distinctly separated by submucosal layer. In case of dimethylhydrazine treated colon, mucosal epithelial layer appeared to be discontinuous with occasional region of un-uniform layer of morphologically different cells (identified as neoplastic cells responsible for colon adenocarcinoma) or few propagations consisting of different type of cells. These regions were crowded with irregular cells with large morphological variations^22,23^. In some sections, cancerous regions have been found to infiltrate into the submucosal layer towards muscular layer. On the other hand, **2c-NP** treated animals with colorectal cancer showed a significant reduction in propagation of cancerous regions, restoring of epithelial layer and disappearance of ulcerative regions. This demonstrates the effectiveness of **2c-NP** for treating colorectal cancer. Interestingly, **2c-NP** administration in normal animals was not found to alter histopathological integrity of normal colon (Fig. 11i,j), suggesting **2c-NP** is not toxic to normal colon cells.

## Discussion

One of the major challenges among the scientific community is to develop safe and effective anticancer drugs. Nanotherapeutics have the ability to reduce cytotoxicity of anticancer drugs toward normal cells. Recently, development of polymer-based drug delivery systems (DDS) of nanosize has come under the spotlight due to their recognised ability to provide maximum efficacy by reducing drug degradation, reducing adverse effects by providing sustained release of the therapeutic agent within a threshold concentration level, and triggering site specific action due to preferential uptake and retention in cancer cells^24,25^.

In this study, owing to the impressive biological activities such as anti-inflammatory, anti-bacterial, anti-malarial, and anti-proliferative of betulinic acid analogue **2c**^26–29^, the drug was encapsulated into PLGA to improve its scope of application in cancer therapy. Initially, we conducted FTIR analysis of each formulation ingredient, their physical mixture, blank formulation, and **2c** loaded formulation to understand their physico-chemical compatibility. Minor shifting of non-characteristic peaks of **2c** in drug loaded formulation (**2c-NP**) may be attributed to weak hydrogen bond formation and electrical attraction such as dipoles moment, van der Waals force). These types of bond formations could inhibit crystallisation of **2c** inside the formulation facilitating its presence in the amorphous state, which may give rise to sustained drug release and prolonged bioavailability of **2c** from **2c-NP**. However, the presence of all characteristic peaks of **2c** in **2c-NP** suggests chemical integrity of **2c** inside the formulation, which is important to exert adequate biological activities upon its release from the nanoparticles.

Surface morphology of the formulated nanoparticle has crucial importance in drug uptake by cells or organs as there is an interaction of the nanoparticle surfaces with biomembrane when they are exposed to the biological system. Morphological analysis through FESEM, AFM, and cryo-TEM proved formulation of spherical, aggregation-free, stable nanoparticles. The sizes of most of the particles were found to be within 200 to 500 nm (Supplementary Fig. S1). This fulfills the initial criteria to deliver **2c-NP** as a targeted nanoformulation. The negative zeta potential (due to the presence of –COOH group in the polymer) was −8.94 mV which is within the range of −30 to +30 mV suggesting that **2c-NP** is prone to sedimentation in suspension on prolonged storage, and hence needs to be stored as lyophilised powder and reconstituted in dispersed form before use^11^.

The *in vitro* release kinetic study revealed that the drug released from the formulation in a sustained manner. The data of *in vitro* drug release pattern of the nanoformulation followed Higuchi model of kinetics. The regression coefficient (R^2^) value indicated good linearity (0.9209, Supplementary Table T2). Drug release data further suggests that there was an initial burst release of drug from **2c-NP**, which might provide fast initial drug concentration in blood^30^.

*In vitro* cytotoxicity analysis through MTT assay revealed a significant reduction in IC_50_ value (Fig. 4), evincing better efficacy at low concentration as well as possible reduction of side-effects. While delivering drug molecules through nanoformulation, the target-specific cellular internalization is a major subject of concern. The normal physiological system considers the micron size particulate formulations as ‘foreign particles’ and normally eliminates them by phagocytosis^31^. But nanoparticles are not recognised in general by macrophages and can remain in blood stream for considerably longer time period^31^. Moreover, they can be pinocytosed inside macrophages which is an extracellular concentration dependent phenomenon^31^. Confocal microscopic analysis (Fig. 5a) demonstrated predominant accumulation of fluorescent labeled nanoparticles (FITC-**2c-NP**) in the perinuclear region, testifying to the ability of **2c-NP** to reach nucleus.

It has been already reported that biodegradable PLGA is mostly safe for various kinds of cells, the safety depending on concentration of nanoparticle and exposure time^32,33^. Xiong *et al*. (2013) studied the cytotoxicity of PLGA nanoparticle on normal cells, focusing on the metabolic activity of macrophages^34^. They showed that the release of TNFα was negligible from phagocytic cells (RAW264.7 and BEAS-2B) when treated with PLGA nanoparticle of 200 nm size range, up to the concentration of 300 µg/ml. The nanosize range of **2c-NP** might facilitate intracellular uptake of **2c-NP** in HT-29 cells^31^.

To explore possible mechanistic pathway of cell death induced by **2c-NP** in HT-29 cells, we concentrated on the intrinsic pathway of apoptosis as evidenced by our previous report^7^. Mitochondrial membrane depolarisation is a hallmark of apoptosis where the mitochondrial membrane becomes permeable to cytochrome-c, resulting in release of internal cytochrome-c in the cytosol. This cytochrome-c can activate caspases and trigger apoptosis^35^. After treating HT-29 cells with **2c-NP**, induction of apoptosis was found to increase predominantly (Fig. 7b) compared to free drug treatment^7^. The data have suggested efficient depolarization of mitochondrial membrane in HT-29 cells upon increasing cellular uptake of **2c-NP**. The percentage of apoptotic cells after treatment was found to increase in a time dependent manner.

In order to get more insight into the possible mechanism of greater cytotoxic effect of **2c-NP**, cell cycle analysis was conducted. Apoptosis and cell cycle are interlinked phenomenon through the involvement of some genes such as E2F, p53 and RB^36^. The nanoparticle mediated S-phase arrest indicates the efficacy of **2c-NP** in blocking DNA synthesis in HT-29 cells which well correlates with the potent inhibitory role of betulinic acid and its derivatives toward topoisomerase II^37,38^. DNA fragmentation and chromatin condensation also supported this phenomenon as evidenced by confocal images the treated cells (Supplementary Fig. S4). In this context, the key regulatory gene p53 plays a crucial role by facilitating the passage of cells from late G1 phase to S phase^18^. Additionally, p53 restoration promotes the down-regulation of anti-apoptotic gene Bcl-2, thereby ensuring the onset of apoptosis^39^. Our data demonstrate that compared to untreated cancer cells, **2c-NP** treatment of HT-29 cells enhanced S phase arrest of the cell cycle in a time dependent manner, indicating reactivation of p53. Figure 8 revealed a time dependent increase of p53 expression level in three different colorectal cancer cells, HT-29 (most predominant), HCT-15 and HCT-116 upon incubation with **2c-NP** for 24 h and 48 h. Apart from the well-known regulation of apoptotic pathway of cell death, p53 can induce oxidative stress-induced necroptosis^18^. In our experiments caspase-8 activity has increased 4.3 times in 6 h upon **2c-NP** treatment. This ensures dominancy of apoptosis in p53 expression over necroptosis in **2c-NP** induced cell death. Interestingly, overexpression of caspase-3 (∼5.5 fold increase compared to control) and caspase-9 (∼12.6 fold increase compared to control) ensures mitochondrial pathway dependent apoptotic cell death^7^. We therefore analysed caspase 8 activity in **2c-NP** treated cells. Expression of caspase-8 has an inhibitory action in necroptosis initiation. Up-regulation of Caspase-8 inhibits formation of necrosome by cleaving RIPK1 (Receptor-interacting serine/threonine-protein kinase 1), which forms microfilament like structures known as necrosome after binding with RIPK3^40^.

The staining of nucleus (DNA) by acridine orange/ethidium bromide distinctly separated normal and cancer cells, and those undergoing apoptosis after treatment with **2c-NP**. The apoptotic cells appeared with orange or yellowish orange nuclei^41,42^, but the difference between early and late apoptotic cells is not so prominent in the figures. The live cells are exclusively green as ethidium bromide is impermeable to live cells. The data supported the outcome of apoptosis assay by FACS analysis in a quantitative way.

To evaluate *in vivo* efficacy of nanoformulated **2c**, biodistribution and radio imaging study have been conducted using ^99m^Tc radiolabeled **2c-NP**. The data reflect predominant accumulation of **2c-NP** in the large intestine in DMH treated rats. Accumulation of **2c-NP** in colon (measured as activity present in whole intestine) reached highest at 5 h. The intensity of radiation in intestinal region was higher at 5 h than at 2 and 1 h (Fig. 9b). Upon observation of dissected colons of CRC animals, under gamma camera it was observed that within the same tissue, distribution of radioactivity is not uniform (identified by intensity differences in Supplementary Fig. S7b,d). This may ensure development of different affected regions (CRC) in the colons. As the nanoparticles accumulate predominantly in tumor regions due to enhanced permeability and retention effect, the proportionate difference in radioactivity supports presence of carcinoma lesions in those regions of colon.

^**99m**^**Tc-2c-NP** level in blood was significantly high throughout the study time, indicating good amount of blood retention of **2c-NP** and stability of the radio-complex *in vivo*.

Liver accumulation of radiolabeled nanoparticles was initially high and decreased gradually with the progress of time (Table 1). The polymeric nanoparticles were first accumulated in liver within a short time of injection but a significant number of nanoparticles passed out rapidly from hepatic endoplasmic reticulum due to its nanosize range. As liver might be capable of digesting a portion of the entrapped nanoparticles, the amount of radioactivity in liver decreased with the progression of time. But amount of radioactivity in stomach was almost the same throughout the time of the experiment which assured stability of ^**99m**^**Tc-2c-NP** complex in stomach.

Accumulation of ^**99m**^**Tc-2c-NP** in heart was significantly low, suggesting low possibility of cardiac side-effects after administration of ^**99m**^**Tc-2c-NP**. Although the ratio of tumor/blood radioactivity increased with time, the ratio of radioactivities in muscle/blood was almost the same throughout the study period and was significantly (p < 0.05) low. This suggests high tumor selectivity of ^**99m**^**Tc-2c-NP** instead of aberrant accumulation in muscle. The major way of elimination of radiolabeled nanoparticle was found to follow the urinary route. The low half-life of ^99m^Tc (~6 h) limited the *in vivo* tracking of **2c-NP** for a prolonged time but it provided valuable information regarding the transport behaviour of **2c-NP** in tumor bearing live animals.

In case of both normal and tumor bearing colon (Fig. 10) accumulation of FITC-labeled **2c-NP** was considerably higher at 5 h than at 2 h. In case of tumor bearing colon significant part of epithelial layer was disrupted due to cancer development and propagation. Despite this, accumulation of **2c-NP** was higher compared to normal colon (as suggested by more fluorescence in those regions) as reflected clearly by the image at 2 h. This implies the specificity of **2c-NP** toward cancer tissues, and may be explained by enhanced permeability and retention (EPR) effect. In case of normal colon each different tissue layer (e.g., mucosal, sub-mucosal, muscular layer) was found to be distinct and aligned with different types of homogeneous cells (among which goblet cells were predominantly visible). But in case of DMH treated animals, the most affected part in colon was the epithelium, where cancer development produced numerous heterogeneous cells which were morphologically different from the cells of normal colonic tissues.

Histological observation gathered more information about tumor generation in colon following DMH treatment and efficacy of **2c-NP** to inhibit tumor propagation. In normal colon all the cells of mucosal and sub-mucosal layer appeared with morphological similarity (Fig. 11a,b). As the duration upon treatment with DMH progressed, the mucosal epithelium became heterogeneous due to generation of cancerous cells and their abnormal growth followed by infiltration in sub-mucosal layer (Fig. 11c–f). Treatment with **2c-NP** was able to significantly restore the epithelial homogeneity, suggesting its theranostic effect in tumor inhibition in a curative manner^43^.

DMH-induced colorectal cancer bearing animals treated with **2c-NP** experienced significant tumor reduction and distinctive cure of the ulcerative regions of colon. We reckon that this happened due to the increased and sustained bioavailability of **2c**, and neoplastic cell specific accumulation when delivered as **2c-NP**. Nanoencapsulation of **2c** caused an appreciable decrease in cytotoxicity in normal cells *in vitro* and improvement of *in vivo* therapeutic efficacy. Overall the study reflects that **2c-NP** could be explored for future development of pharmaceuticals to combat colorectal cancer, as it was capable of more specific delivery of the drug to the neoplastic colon cells and inducing apoptosis therein, as distinct from its function in normal cells.

## Materials and Methods

### Materials

Poly (D,L-lactic-co-glycolic acid) (PLGA) (75:25), succinic acid, didodecyl dimethyl ammonium bromide (DMAB) and 1,2-dimethylhydrazine (DMH) were purchased from Sigma Aldrich (St. Louis, MO, USA). 3-(4, 5-Dimethylthiazol-2-yl)-2, 5-diphenyltetrazolium bromide (MTT) was purchased from USB Corporation (USA). Dulbecco’s Modified Eagle Medium (DMEM) (High glucose), Eagle’s Minimum Essential Medium (EMEM), heat inactivated fetal bovine serum (FBS) and 5,5′,6,6′-tetrachloro-1,1′,3,3′- tetraethyl benzimidazolylcarbocyanine iodide (JC-1) were purchased from Invitrogen (Carlsbad, CA, USA). Polyvinyl alcohol (PVA, MW 125,000) was obtained from S.D. Fine-Chem. Ltd. (Mumbai, Maharashtra, India). Fluorescein isothiocyanate (FITC) was purchased from HiMedia Lab. Pvt. Ltd. (Mumbai, Maharashtra, India). All the chemicals were of analytical grade or molecular biology grade and used as received without any further purification.

### Animal model development

Seventy Sprague-Dawley rats (Body weight = 140–160 g) and eighty Swiss albino mice (Body weight = 20–25 g) of either sex (Male:Female = 1:1) were procured from National Institute of Nutrition (Hyderabad, India). They were maintained in polypropylene cages and housed at 25 °C ± 1 °C temperature accompanied with a relative humidity 55% ± 5% and a normal day and night photoperiod for at least 2 weeks before conducting any experiments. Animals were primarily divided into four groups named Normal control, Carcinogen control, Carcinogen treated and Normal treated animals. The detailed information of each group has been described in supplementary section (Supplementary Table T3). Animals were further subdivided according to experimental requirement as described in respective methodology section. For both rats and mice a normal control group was maintained by nourishing with regular foods and in addition the other animals were used to generate colon cancer model by treating them with dimethylhydrazine (DMH). In case of mice DMH was injected intraperitoneally at a dose of 20 mg/kg body weight once in a week for 12 weeks whereas in case of rat intraperitoneal dose of DMH was 25 mg/kg body weight once in a week for 9 weeks. The complete experimental procedures were approved by Animal Ethics Committee (AEC), Jadavpur University (JU-AEC; Registration Number: 1805/GO/Re/S/15/CPCSEA) and the guidelines of Purpose of Control and Supervision of Experiments on Animals (CPCSEA) were carefully followed in all the experimental processes. *In vivo* studies and experimental protocols were approved by JU-AEC (Protocol approval. no.: AEC/PHARM/1701/3/2018).

## Methods

### Drug-excipient interaction study

Drug-excipient interaction was studied by Fourier-transform infrared (FTIR) spectroscopy to identify presence of any interaction between the drug and the selected excipients^11^. A set of seven samples were chosen for analysis and they include blank nanoparticle (nanoparticle without drug); drug (BA analogue, **2c)**; polyvinyl alcohol (PVA); poly-lactide-co-glycolide (PLGA); physical mixture comprising PLGA and PVA; **2c** loaded PLGA nanoparticle (**2c-NP**) and physical mixture comprising **2c**, PLGA and PVA. Each sample (5 mg) was mixed up with IR grade KBr at 1:100, ratio and punched to make pellets. The pellets were then scanned in a FTIR instrument (Bruker FTIR, Tensor- 27) in a wavelength range of 4000–400 cm^−1^.

### Preparation of PLGA nanoparticles containing lead betulinic acid analogue (2c-NP)

Multiple emulsion solvent evaporation technique was employed to prepare PLGA nanoparticles with some definite modifications^11^. Briefly, 5 mg of lead betulinic acid analogue **2c**, and 50 mg of PLGA (75:25) were dissolved in 3 ml of organic phase (DCM:Acetone :: 4:1 v/v). Primary emulsion was prepared by emulsifying 1.5 ml of 2.5% aqueous PVA with the above-mentioned organic phase by homogenization for 3 min, using a high speed homogenizer at 20,000 rpm (IKA Laboratory Equipment, Model T10B, Ultra- Turrax, Staufen, Germany). A primary emulsion thus obtained was further emulsified with 1.5% aqueous PVA solution (75 ml) by homogenizing for about 8 min at 20,000 rpm using the same instrument. The double emulsion was then sonicated in a bath-sonicator for 30 min for size reduction of emulsion droplets. The final double emulsion was then stirred overnight on a magnetic stirrer at room temperature to remove traces of organic solvents. Nanoparticles were formed during evaporation of organic solvents and subsequent solidification of polymers. Then the nanoparticles were separated by two consecutive centrifugation steps (once at 5,000 rpm for 10 min followed by 16,000 rpm for 45 min) in a cooling centrifuge (Hermle refrigerated centrifuge, Siemensstr, Wehingen, Germany). Excess PVA associated with nanoparticles was washed out by washing **2c-NP** with double distilled water two times and precipitated by centrifugation at 16,000 rpm/min. The final pellet obtained was redispersed in 2 ml of double distilled water, frozen in a refrigerator (So-Low, Environmental Equipment, Ohio, USA) and was lyophilized in a Freeze dryer (Laboratory Freeze Dryer, Instrumentation India, Kolkata, India) to get in a dry powder form. Nanoparticles loaded with FITC were prepared by adding 100 μl of 0.4% (w/v) ethanolic FITC solution into the initial organic phase during the preparation of primary emulsion.

### Determination of particle size distribution and zeta potential

Dynamic light scattering method was utilized to determine average particle size and zeta potential distribution of nanoparticles obtained. Weighed amount of the **2c-NP** was dispersed in Milli-Q water (Millipore Corp., Billerica, MA, USA) through a cycle of brief sonication and vortexing and then analyzed in Malvern Zetasizer Nano-ZS 90 (Malvern Instruments, Malvern, UK) for particle size and zeta potential measurement^11^.

### Drug loading (%) and entrapment efficiency (%) analysis

Drug loading (%) was determined by extracting the drug (2c) material from the nanoparticles into solvent mixtures, followed by spectrophotometrical analysis^11^. Weighed amount of lyophilized nanoparticle formulation (2 mg) was solubilized in 2 ml of acetonitrile:water :: 85:15 v/v mixture and agitated in an incubator shaker (Somax Incubator Shaker; Shenjhen Pango Electronic Co. Ltd., China) for 4 h. The suspended nanoparticles were separated by cold centrifugation at 16,000 rpm for 15 min and the clear supernatant was analysed spectrophotometrically to measure the drug content in it. Drug loading was determined using the following equation:

3 $${\bf{D}}{\bf{r}}{\bf{u}}{\bf{g}}\,{\bf{l}}{\bf{o}}{\bf{a}}{\bf{d}}{\bf{i}}{\bf{n}}{\bf{g}}\,({\bf{a}}{\bf{c}}{\bf{t}}{\bf{u}}{\bf{a}}{\bf{l}})\,( \% )=\frac{{\rm{Amount}}\,{\rm{of}}\,{\rm{drug}}\,{\rm{in}}\,\mathrm{nanoparticles}\,}{{\rm{Amount}}\,{\rm{of}}\,{\rm{nanoparticles}}\,{\rm{obtained}}}\times 100$$

Entrapment efficiency was calculated using the following Eq. (2):

4 $${\bf{E}}{\bf{n}}{\bf{t}}{\bf{r}}{\bf{a}}{\bf{p}}{\bf{m}}{\bf{e}}{\bf{n}}{\bf{t}}\,{\bf{e}}{\bf{f}}{\bf{f}}{\bf{i}}{\bf{c}}{\bf{i}}{\bf{e}}{\bf{n}}{\bf{c}}{\bf{y}}\,( \% )=\frac{\mathrm{Drug}\,\,{\rm{loading}}\,({\rm{actual}})\,( \% )}{{\rm{Drug}}\,{\rm{loading}}\,({\rm{theoretical}})\,( \% )}\times 100$$

### Analysis of nanoparticle morphology

Field emission scanning electron microscopy was used to understand morphology of dry lyophilized nanoparticles. Appropriate amount of **2c-NP** was mounted on a carbon stub followed by coating with a gold layer (around 4 nm thickness) and observed under a field emission scanning electron microscope (JEOL JSM-7600F, Japan) at 20,000 × and 40,000 × magnifications. AFM was utilized to understand particle morphology of **2c-NP**s under hydrated condition. **2c-NP**s were dispersed in MilliQ water to attain 50 µg/ml concentration and filtered once through 0.22 µm filter to eliminate any preformed large aggregates. 4 µl of this filtered dispersion was placed on a cleaved mica sheet and dried in air at room temperature for 20 min. When water was evaporated forming a thin transparent layer on mica sheet, it was observed under an AFM instrument (5500 Agilent Technologies, Santa Clara, CA, USA) in tapping mode^19^. Cryo-transimission electron microscopy was used to understand internal structure of **2c-NP**. Nanoparticles were dispersed in MilliQ water to attain approximately 30 µg/ml concentration and a volume of 4 µl from this solution was placed on a glow-discharged carbon-coated copper grid of 300 mesh size. Then it was plunge-frozen in liquid ethane and excess liquid was removed with specially designed filter paper using Vitrobot instrument (FEI Company, USA). The copper grid was then transferred in a cryoholder and observed in Technai POLARA (FEI Company, Oregon, USA) electron microscope, empowered by a 300 kV field emission gun (FEG). Images were captured in a 4k × 4k CCD camera^44^.

### *In vitro* release kinetics

Drug release behavior from **2c-NP** was studied in phosphate buffered saline (PBS)^11,45^. **2c-NP** suspension (1 mg/ml, 2 ml) was prepared in PBS (pH 7.4) and was agitated in an incubator shaker (Somax Incubator Shaker; ShenjhenPango Electronic Co. Ltd., Shenzhen, China) at 37 °C. The suspension (1 ml) was withdrawn from this vial at different time intervals (1 h, 2 h, 4 h, 8 h, 24 h, 48 h, 72 h, 144 h, 168 h, 360 h, 432 h, 456 h, 480 h, 504 h, 648 h, 672 h, 696 h, 960 h, 1032 h, 1200 h, 1440 h, 1680 h, 1920 h, 2160 h), centrifuged and the supernatants were analyzed for drug content using UV-visible spectrophotometer^11^. For each sample withdrawal the pellet obtained from centrifugation was redispersed in 1 ml of fresh PBS and replaced into the vial. A cumulative % drug release *vs* time graph was generated using a previously made calibration curve. Different kinetic models like first order, zero order, Korsmeyer-Peppas, Highuchi, and Hixson-Crowell models were used to interpret the release profile of **2c** from **2c-NP**^46^.

### Cell lines and culture maintenance

The adherent colorectal cancer cell lines HT-29, HCT-116 and HCT-15 as well as normal human embryonic kidney cell line (HEK 293) were collected from National Centre for Cell Science (NCCS), Pune, India; and then maintained in high glucose DMEM medium (supplemented with 10% FBS, 50 IU/ml penicillin G and 50 µg/ml streptomycin) in a humidified incubator under 5% CO_2_ environment. Normal colon cell line (CCD-33Co) procured from American Type Culture Collection (ATCC), Manassas, Virginia, USA, was maintained in EMEM medium with the same other conditions mentioned above. Sub-culturing was done after every 90% confluence. Trypan blue exclusion method was used to determine cell viability whenever necessary.

### *In vitro* cytotoxicity assay using MTT

The cytotoxic effect of free **2c** and **2c-NP** upon colon cancer cell line HT-29 was quantified through MTT assay. HT-29 cells (1.25–2.5 × 10^4^) were seeded in the wells of a 96 well plate and incubated with supplemented DMEM media overnight at 37 °C under 5% CO_2_ environment. Thereafter the media was removed and the cells were treated with media containing free **2c** suspension (200 µl, concentration range 1–50 µmol) and **2c-NP** suspension (200 µl, equivalent **2c** concentration range 1–50 µmol) in two different groups for 48 h. In case of control group, the cells were not treated with drug. After completion of treatment time 20 µl of MTT solution (5 mg/ml in PBS) was added in each well and incubated for 3–4 h at 37 °C. The MTT solution was then removed and 100 µl of DMSO was added in each well and rotated in a shaker for 15 min to extract the formazan crystals from the cells, formed in the reaction of MTT with mitochondrial reductase enzyme. The plate was then analyzed in an ELISA reader (Bio Rad, CA, USA) at 540 nm. Cell viability % was plotted against **2c**/**2c-NP** concentration and IC_50_ value was determined.

The significance of the results expressed a IC_50_ value was determined using graphical extrapolation using Graph Pad Prism software (version 5, Graph Pad Prism software Inc, San Diego, CA, USA)^7^.

### *In vitro* cellular uptake study

Intracellular uptake of **2c-NP** was studied *in vitro* using HT-29 cells by confocal laser microscopy and quantified by flow cytometry. For confocal laser microscopy, around 10^4^ cells were seeded on a cover slip, already placed in a 35 mm tissue culture dish with media and incubated at 37 °C overnight. The cells were treated with IC_50_ concentration (11.8 µM) of FITC labeled nanoparticles in different groups (1 h, 2 h and 4 h). After completion of treatment the cells were washed with PBS, fixed using 70% ethanol, co-stained with DAPI (for nucleus) and mounted on a slide to observe under confocal microscope (Olympus FluoView FV10i, Olympus). Filters of FITC (Ex/Em 495/519 nm) and DAPI (Ex/Em 359/461 nm) were used to capture dual color images^47,48^.

Flow cytometry was used for determining time dependent intracellular uptake of PLGA nanoparticles. Briefly, 10^6^ HT-29 cells were placed in a set of 60 mm diameter tissue culture dishes with complete media (different plates for different point, i.e. 1 h, 2 h, 4 h) and incubated overnight at 37 °C. Then the cells were treated with FITC loaded nanoparticles except the control group. After completion of treatment, the treatment solution was removed, the cells were detached from the plates, suspended in FACS tubes to analyze in a flow-cytometer (BD LSRFortessa^TM^, BD Biosciences, San Jose, USA). The data were acquired through FACS Diva software (BD Biosciences)^49^.

### Detection of cell death by acridine orange/ethydium bromide dual staining

Cell death induced by **2c-NP** was observed in confocal laser microscopy after treating HT-29 cells with IC_50_ concentration of **2c-NP**. Briefly, 5 × 10^4^ cells were seeded in each cover slip placed in 35 mm cell culture dishes. The cells were treated with **2c-NP** at its IC_50_ concentration for 12 h, 24 h and 48 h; except the control group which received media only. Thereafter the media were removed and the cells were incubated with acridine orange (4 µg/ml) and ethidium bromide (4 µg/ml) for 3 min^50^. The cells were washed with PBS and mounted on slide. They were then observed in confocal laser microscope (Olympus FluoView) using the filters of acridine orange DNA (green) (Ex/Em 502/526 nm) and propidium iodide (Ex/Em 537/619 nm).

### Mitochondrial membrane depolarization analysis using JC-1

Mitochondrial membrane depolarization also suggests induction of apoptosis^7^. In case of apoptosis mitochondrial membrane becomes depolarized (more positive) which causes a significant change in transmitochondrial potential difference. This was analyzed using 5,5′,6,6′-tetrachloro-1,1′,3,3′-tetraethylbenzimidazolyl-carbocyanine iodide (JC-1) following standard protocol. Briefly, 1 × 10^6^ HT-29 cells were harvested in 60 mm dishes, incubated overnight in a humidified incubator followed by treatment with **2c-NP** for required time periods (12 h, 24 h and 48 h). Thereafter the cells were detached from the dishes and incubated with 10 µl of 200 µM JC-1 in 1 ml complete media for 10 min at 37 °C in dark. The media was removed by centrifugation; the cells were suspended in buffer and analyzed in a FACS instrument^51^.

### Apoptosis assay

To quantitatively measure the ability of **2c-NP** to trigger apoptosis and/or necrosis in HT-29 cells, apoptosis assay was performed using standard protocol. Briefly, HT-29 cells (2.5 × 10^5^/ml) were incubated with IC_50_ concentration of **2c-NP** (11.8 µM) for 12 h, 24 h, and 48 h at 37 °C under 5% CO_2_ environment. In case of control group, the cells were not treated with drug. At the end of treatment time cells were detached with trypsin, washed by PBS (2×) and resuspended in 100 µl of Annexin V binding buffer (1X) (10 mM HEPES, 140 mM NaCl, 2.5 mM CaCl_2_; pH 7.4). Annexin V-Alexa 568 (2 µl) was added to cell suspension, incubated in dark for 15 min and diluted with additional 400 µl binding buffer (1X). PI (5 µl, 50 µg/ml) was added just before analysis^9^. Data was obtained using a FACS Aria flow cytometer (Becton Dickinson, USA) using channels of FITC (excitation/emission 488 nm/530 nm) and PE-Texas red (excitation/emission 561 nm/616 nm) and post capturing analysis was done with BD FACS Diva software (Becton Dickinson, USA)^8,52,53^.

### Effects of 2c-NP on the cell cycle of HT-29 cells

To investigate probable mode of action of **2c-NP**, its effect on cell cycle was analyzed using Hoechst 33258 live cell staining method. Briefly, **2c-NP** treated cells were incubated with Hoechst 33258 (5 µg/ml final concentration) for 1 h and then analyzed by flow cytometry using the filter Indo-I (Violet-A) in FACS instrument. Hoechst 33258 (5 µg/ml) was added to the cells to stain DNA and analyzed by flow cytometry^54^.

### Nuclear morphology analysis

Hoechst stain was used to visualize nuclear morphology under fluorescence microscope^8^. Briefly, 2.5 × 10^4^ HT-29 cells were treated with **2c-NP** (IC_50_ = 11.8 µM) for 12 h, 24 h and 48 h. After removing the treatment solution the cells were washed with cold PBS, fixed with 4% paraformaldehyde at 4 °C for 20 min and washed again to remove excess paraformaldehyde. Hoechst 33258 (5 µg/ml final concentration) was added to the cells and incubated in dark at 4 °C for 30 min. Thereafter cells were washed three times with PBS and spotted onto poly-L-lysine coated slides with 50% glycerol as mountant. The slides were then observed under a fluorescence microscope (Leica TCS SP2 System Leica Microsystem, Heidelberg, Germany, 10X) to find out morphological changes of cells for the treatment with **2c-NP**.

### Study of p53 expression level

Expression of p53 was observed by confocal laser microscopy after labelling with rabbit anti-p53 primary antibody followed by staining with Alexa Flour 546 coupled goat anti-rabbit IgG secondary antibody. HCT-15, HCT-116 and HT-29 cells were treated separately with **2c-NP** at IC_50_ concentration for 24 h and 48 h. In case of control group, the cells were not treated with drug. After removal of treatment solution and washing with PBS, cells were treated with Triton 100X for 10 min to increase the membrane permeability, then exposed to preheated antigen retrieval solution (100 mM Tris, 5% w/v urea, pH 9.5) at 95 °C for 10 min and washed with PBS (3X). Then the cells were incubated overnight with rabbit anti-p53 primary antibody at 4 °C (following the guidelines of the manufacturer). After removing antibody solution, the cells were exposed to blocking solution (1% BSA) for 1 h, washed with PBS and incubated with the secondary antibody (Alexa Flour 546 coupled anti-rabbit IgG) at room temperature for 1 h (according to the manufacturers’ instruction), washed again with PBS and counterstained with DAPI. Finally mounted on slides and observed under a confocal laser microscope using appropriate filters.

### Caspase assay

Expression of p53 is related to both apoptosis and necroptosis^18^ which have been associated with the degree of protein expression. Hence caspase activities expression of caspase-3, caspase-9 and caspase-8 were analysed using enzymatic assay. The enzymatic activity of caspase −3, −9, −8 was assayed in cell lysates (100 µg proteins in 50 µl lysis buffer) using FLICE colorimetric assay kits as per the guideline of manufacturer. Briefly, control and 2c-NP treated (14.9 µm; 12 h, 24, 48 h) cells (2.5 × 10^5^/ml) were washed with cold PBS (4 °C), cell lysates were prepared and subsequently protein concentration was estimated. Lysates were combined with reaction buffer and incubated with specific colorimetric peptide substrates (Ac-IETD-pNA for Caspase-8, Ac-LEHD-pNA for Caspase-9, Ac-DEVD-pNA for Caspase-3; 4 mM, 5 µl) at 37 °C for 6 h. The emission of paranitroanilide (pNA) was measured at 405 nm in an ELISA reader every 30 minutes for 6 h.

### *In vivo* biodistribution in colon cancer bearing Swiss albino mice and Sprague Dawley rats

*In vivo* biodistribution study was carried out in both normal rats (Sprague Dawley) and mice (Swiss albino) as well as rats and mice with colorectal cancer and they are treated with ^99m^Tc radiolabeled **2c-NP**. Briefly, **2c-NPs** were radiolabeled by ^99m^Tc through direct oxidation of acidic SnCl_2_^55,56^. The radiolabeled nanoformulation was injected through tail vein in experimental animals prehydrated by intraperitoneal (administration of 0.9% normal saline before 1 h) route. Radioactivity injected for mice was 12–16 MBq/Kg body weight and for rat it was 14–18 MBq/Kg body weight. Different organs and blood samples were collected from euthanized animals (Five animals for each of the mouse and rat group) at 1 h, 2 h, 5 h post injection in pre-weighed counting vials. Biodistribution of radiolabeled **2c-NP** in different organs and body fluids of interest was analyzed in a gamma scintillation counter (Electronics Corporation of India Limited, Hyderabad, India)^19,55,56^. The data were represented as percent injected dose per g (% ID per g) of tissue or organ.

### Identification of colonic tissue uptake of 2c-NP

The ability of **2c-NP** to reach normal and tumor colonic region was detected qualitatively by confocal microscopy. To observe tumor tissue uptake of nanoparticles in comparison to normal tissue, FITC labeled **2c-NP** suspended in PBS (1 mg/ml) was injected intravenously in normal (rats and mice) as well as rats and mice with colon cancer. The animals were sacrificed at 2 h and 5 h post-injection. The colons were isolated from each animal group (Three animals for each mouse and rat group), rinsed thrice with PBS and kept in 10% formaldehyde for overnight at room temperature. The tissues embedded in paraffin block were sectioned (5 μM thickness) on a microscopic slide, counterstained with DAPI, and observed under confocal laser microscope^21,57,58^.

### *In vivo* radio-imaging

^99m^Tc radioisotope was used to visualize *in vivo* localization of **2c-NP** in colorectal cancer animal models. For gamma scintigraphy study, Swiss albino mice and Sprague Dawley rats were divided into three groups each containing three animals. The experimental animals were anesthetized by intramuscular ketamine hydrochloride injection. In case of Swiss albino mice the radiolabeled **2c-NP** was injected through tail vein (dose 2.5 MBq) and for SD rats it was injected into the femoral vein of one hind limb (preferably left) through a sterile cannula (dose ~37 MBq). Static images were taken at 1 h, 2 h and 5 h post injection under GE Infinia Gamma Camera as head supine position capturing both anterior and posterior view. After the treatment of radiolabeled-2c-NP in carcinogen treated rats and mice, the cancerous tissue portions from the colon were removed at different experimental time points for further confirmation of radiolabeled nanoparticle accumulation in colon. The tissues were visualized under gamma camera and photographed. Then the tissues were processed for histopathological studies to confirm the cancerous growth in the tissue. The images were analyzed by Xeleris Work Station^59^.

### *In vivo* antitumor efficacy of 2c-NP and histopathological analysis of colon

To study the healing efficacy of **2c-NP**, animals treated with colon carcinogen were sub-divided into three groups each containing three animals, one as CRC mice model sacrificed after 7 weeks, second group sacrificed after completion of treatment regimen with DMH (12 weeks) and another treatment group for observing therapeutic efficacy of nanoparticles after treatment with **2c-NP**. To examine the *in vivo* toxicity of **2c-NP**, normal mice were treated with 300 mg/kg of **2c-NP**, i.p dose once in a week for 9 weeks. After completion of treatment schedule (300 mg/kg of **2c-NP** with i.p dose once in a week for 9 weeks) the colons from **2c-NP** treated and untreated CRC mice as well as normal mice treated with **2c-NP** were separated, sectioned on microscopic slides, stained with **H&E** stain and observed under optical microscope to see any histopathological changes due to treatment^60,61^.

### Statistical analysis

All experiments were performed at least three times and in three different sets. All statistical analyses were conducted using one-way ANOVA and graphs were plotted using Graph Pad Prism software (version 5, Graph Pad Prism software Inc, San Diego, CA, USA).

### Ethical conduct of experiments

Permission from the Animal Ethics Committee of Jadavpur University was received before commencing any animal experiments. All experiments have been conducted following the guideline of the Animal Ethics Committee of Jadavpur University.

## Supplementary information

Supplementary Figure S1,Supplementary Figure S2, Supplementary Figure S3, Supplementary Figure S4, Supplementary Figure S5, Supplementary Figure S6, Supplementary Figure S7,
