# Supplementary material for: Nanoencapsulated betulinic acid analogue distinctively improves colorectal carcinoma in vitro and in vivo
Source: Sci Rep. 2019 Aug 8;9:11506. doi: 10.1038/s41598-019-47743-y (PMC6687831; doi:10.1038/s41598-019-47743-y)
Supplement: Supplementary file 1 — Supplementary Figure S1,Supplementary Figure S2, Supplementary Figure S3, Supplementary Figure S4, Supplementary Figure S5, Supplementary Figure S6, Supplementary Figure S7, [file 41598_2019_47743_MOESM1_ESM.pdf]

Nanoencapsulated betulinic acid analogue distinctively improves colorectal carcinoma *in vitro* and *in vivo*

Debasmita Dutta[a], Brahamacharry Paul[c], Biswajit Mukherjee[a]*, Laboni Mondal[a], Suparna Sen[b], Chinmay Chowdhury[b], Mita Chatterjee Debnath [c]

[a] Department of Pharmaceutical Technology, Jadavpur University, Kolkata 700032, India.

[b] Organic and Medicinal Chemistry Division, CSIR- Indian Institute of Chemical Biology, Kolkata 700032, India.

[c] Infectious Diseases and Immunology Division, CSIR- Indian Institute of Chemical Biology, Kolkata 700032, India.

*Corresponding Author

Professor (Dr.) Biswajit Mukherjee

Department of Pharmaceutical Technology,

Jadavpur University, Kolkata-700032, India

Telephone: +91-33-2457 2588

Fax: +91-33-2414 6677

Email: biswajit.mukherjee@jadavpuruniversity.in/ biswajit55@yahoo.com.

Supplementary information

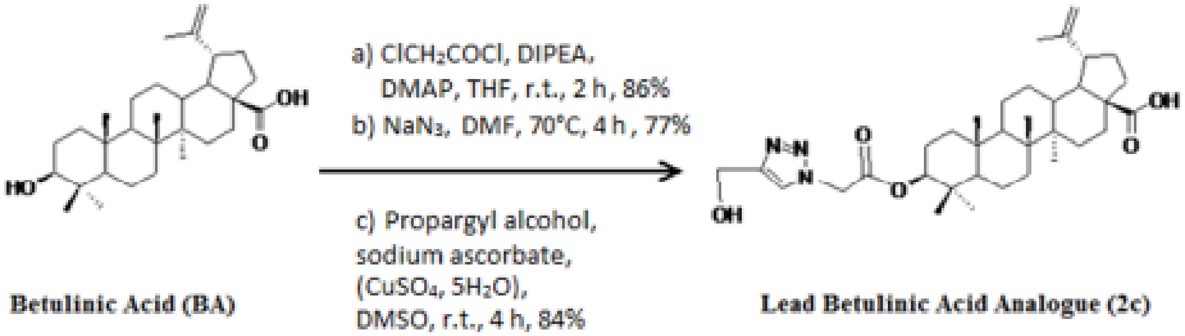

Supplementary Figure S1. Betulinic acid (BA) and the scheme used for synthesis of its bio-active analogue, **2c**.

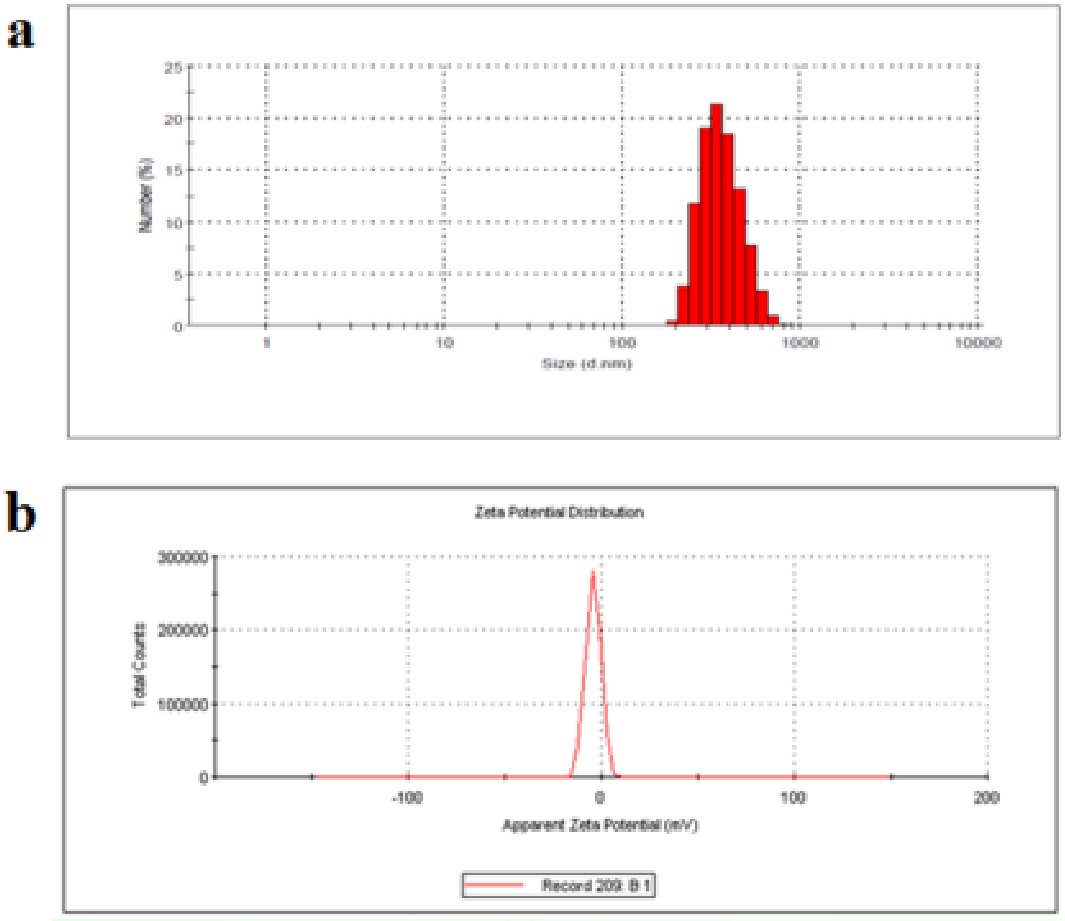

Supplementary Figure S2. Representative data of **(a)** Average particle size distribution and **(b)** zeta potential of nanoformulation, **2c-NP**.

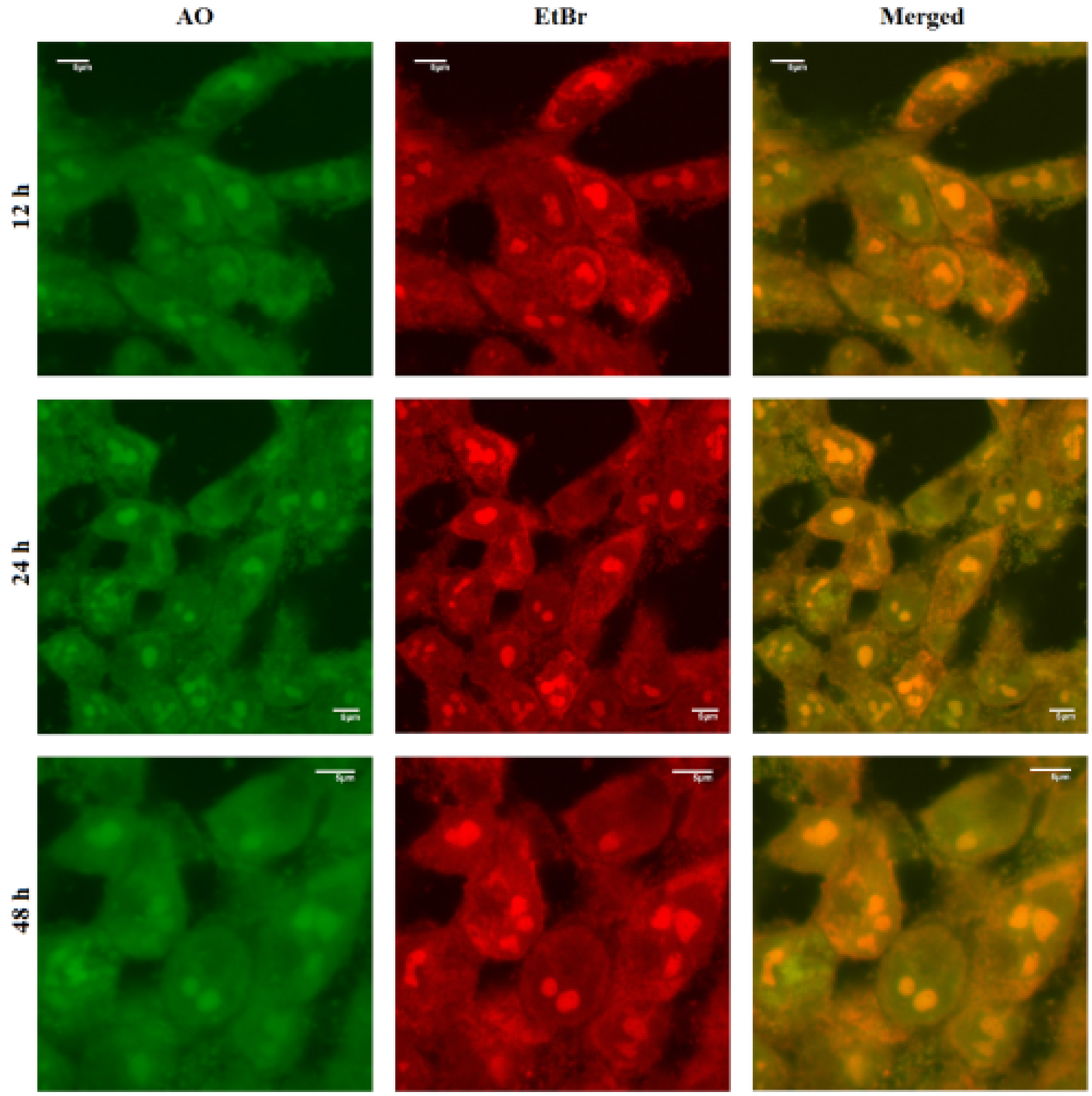

Supplementary Figure S3. Representative image of Acridine Orange/ Ethidium Bromide (AO/EB) staining on higher zooming. Confocal microscopy images of HT-29 cells after treatment with **2c-NP** (11.8 µM; 0–48 h) followed by staining with acridine orange (4 µg/ml) and ethydium bromide (4 µg/ml) captured under Olympus Fluoview 10i confocal microscope at 150X magnification. Scale bar represents 5 µm for 12h, 48h and 10 µm for 24h.

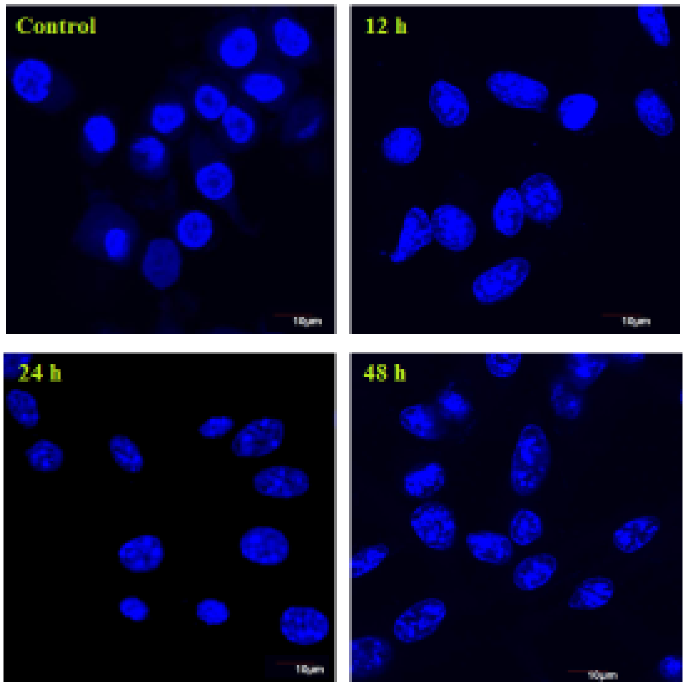

Supplementary Figure S4. Representative image of DNA degradation and apoptotic bodies formation induced by 2c-NP. Control and **2c-NP** (11.8 μM; 0–48 h) treated HT-29 cells (2.5 x 10^4/ ml of RPMI 1640 medium/well) stained with Hoechst 33258 were observed under a Leica confocal microscope (100X). The figure is a representative profile of at least three experiments.

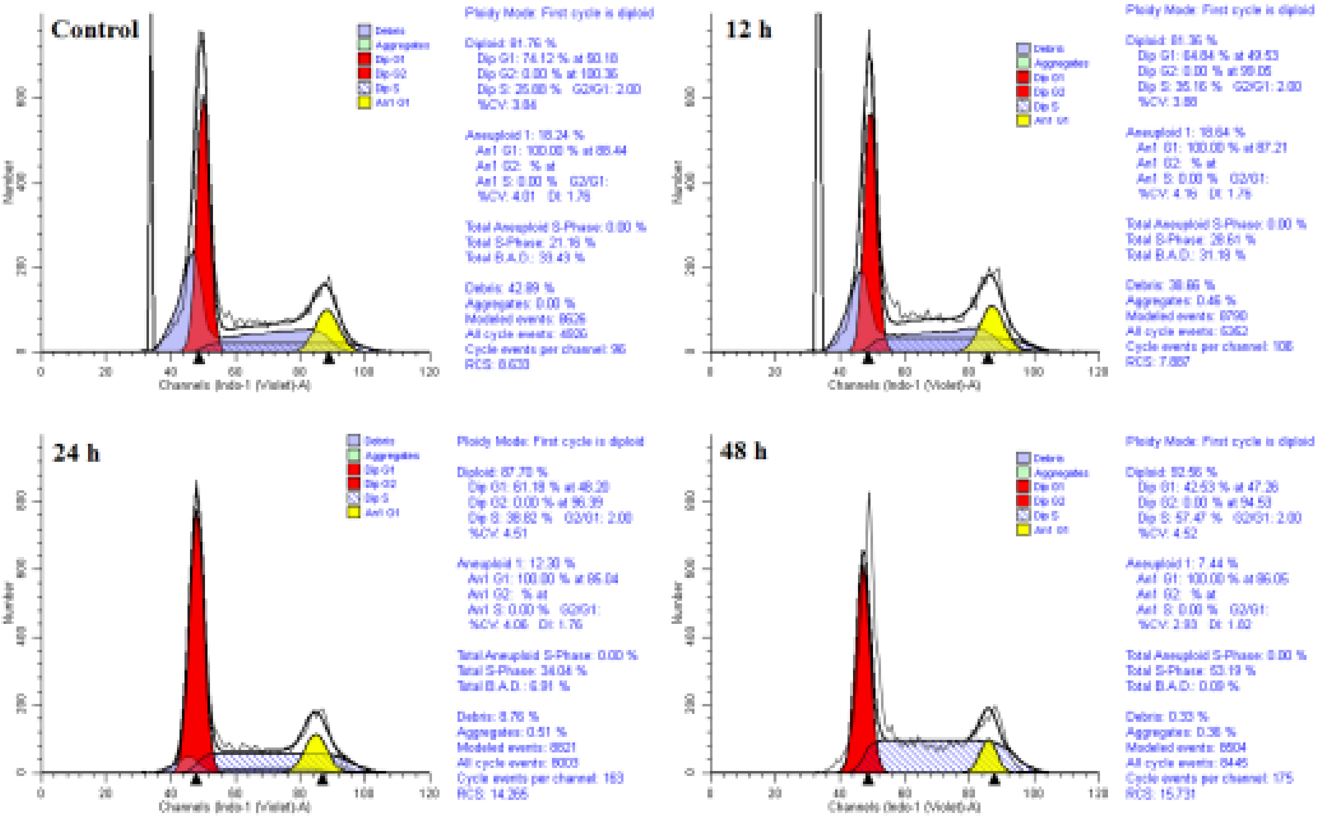

Supplementary Figure S5. Datasheet of cell cycle arrest in HT-29 cells after treatment with 2c-NP for different time points. The data has been analyzed using Modfit software.

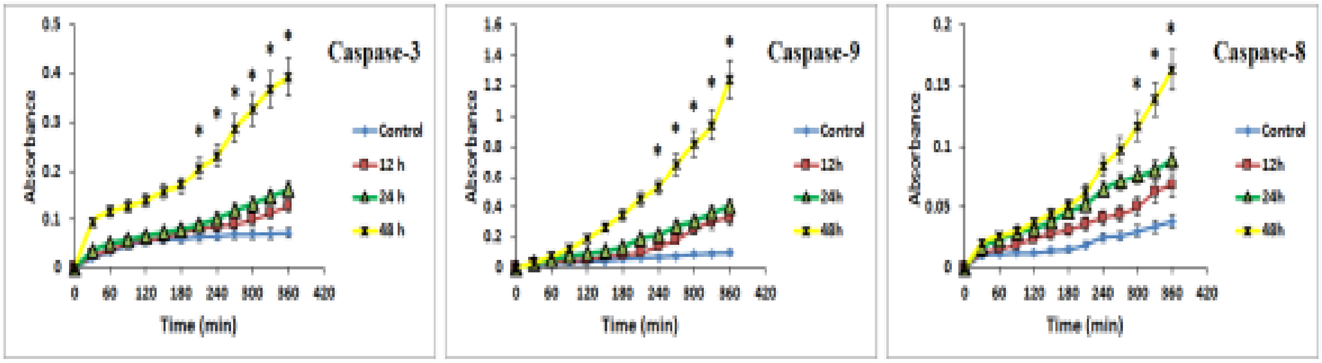

Supplementary Figure S6. Enzymatic analysis of caspase -3, -9 and -8 expression in HT-29 cells after exposure to **2c-NP** for 12 h, 24 h and 48 h.

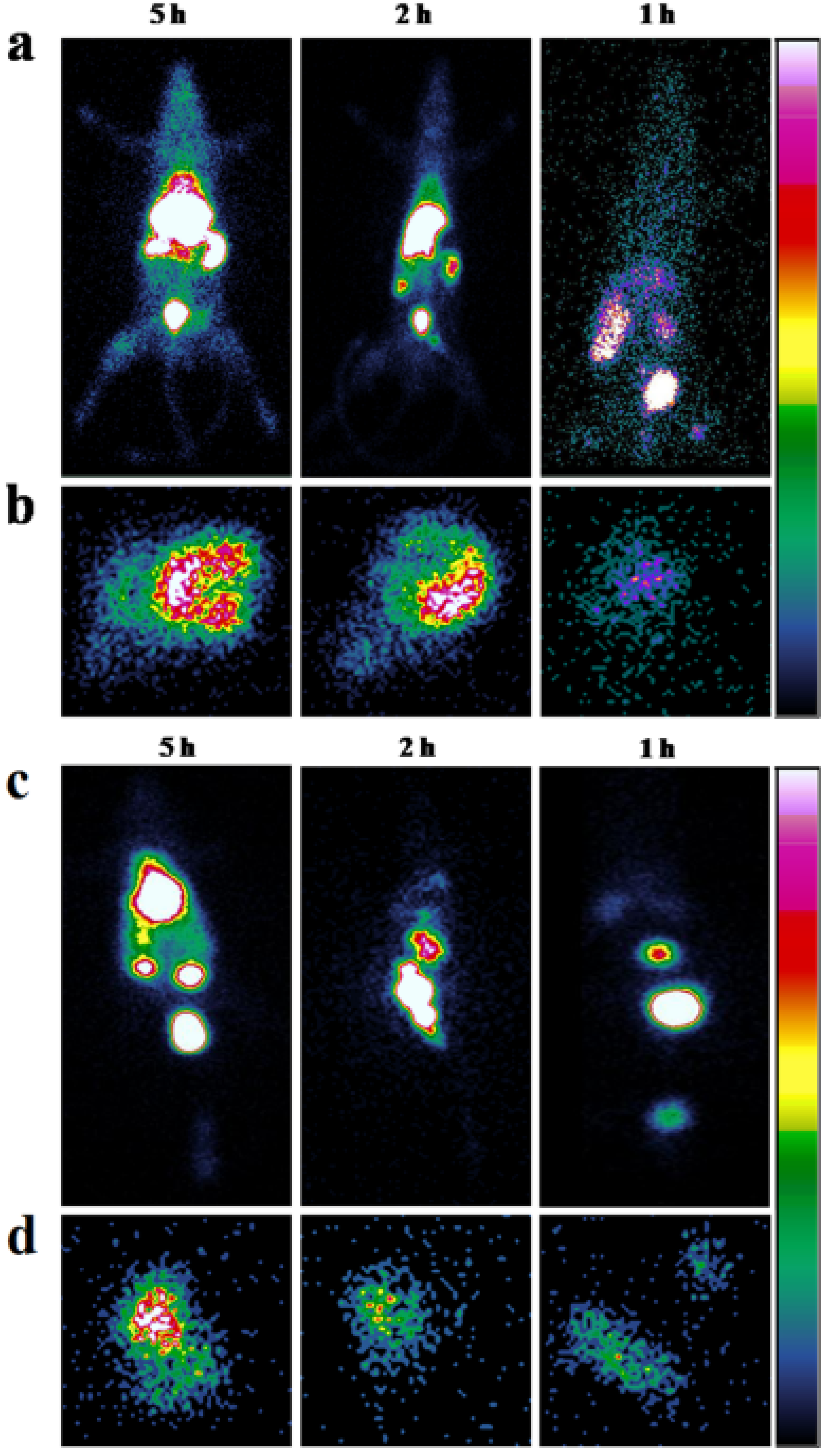
a
b
c
d
5 h
2 h
1 h
5 h
2 h
1 h

Supplementary Figure S7. Representative images of *In vivo* Gamma scintigraphy of CRC animals. (a) Gamma scintigraphic images of Sprague Dawley rats at 1h, 2h and 5h post treatment of ^{99m}Tc – radiolabeled **2c-NP**.(b) Colons isolated from aforementioned animals. (c) Gamma scintigraphic images of Swiss albino mice at 1h, 2h and 5h post treatment of ^{99m}Tc – radiolabeled **2c-NP**. (d) Colons isolated from aforementioned animals.

Supplementary Table T1: IC_{50} values of **2c** and **2c-NP** on different colorectal cancer cell lines and normal cell lines

**Cell Line**	**2c (µM)**	**2c-NP (µM)**
HT 29 (CRC)	14.9 ± 1.3	11.8 ± 1.1
HCT 116 (CRC)	23.8 ± 1.5	19.5 ± 1.1
HCT 15 (CRC)	21.6± 1.2	17.7± 1.6
HEK 293 (Normal kidney cells)	>50	>50
CCD-33-C_O (Normal colon cells)	>50	>50

Supplementary Table T2: Regression coefficient values (R^2) and release exponents as obtained from in vitro drug release data tested on different kinetic models.

***In-vitro* release kinetic models**	**2c-NP**	
	R^2 Value	Representing Equations
Zero Order	0.739	y = 0.0505x + 28.876
First Order	0.8659	y = -0.0005x +1.8478
Higuchi	0.9209	y = 2.0423x + 15.641
Korsmeyer Peppas	0.9028<br>n= 0.403	y = 0.4036 x + 0.808
Hixon Crowell	0.8273	y = -0.0013x + 4.1306

.

Supplementary Table T3: Animal groups and specifications

Animal Group	Number of animals in each group	Specification
Group I - Normal control	Ten rats and ten mice	Received normal food and water *ad libitum*
Group II - Carcinogen control	Forty rats and fifty mice	Received carcinogen with normal food and water *ad libitum*
Group III- Carcinogen treated	Ten rats and ten mice	Received **2c-NP** after carcinogen treatment along with normal food and water *ad libitum*
Group IV – Normal treated	Ten rats and ten mice	Received **2c-NP** along with normal food and water *ad libitum*
